# A single-cell sequence analysis of mouse subcutaneous white adipose tissue reveals dynamic changes during weaning

**DOI:** 10.1038/s42003-024-06448-3

**Published:** 2024-06-29

**Authors:** Shuwen Qian, Chenyang Zhang, Yan Tang, Mengyuan Dai, Zhihui He, Hong Ma, Linyuan Wang, Qiqi Yang, Yang Liu, Wei Xu, Zhao Zhang, Qi-qun Tang

**Affiliations:** 1 Key Laboratory of Metabolism and Molecular Medicine of the Ministry of Education, Department of Biochemistry and Molecular Biology of School of Basic Medical Sciences and Department of Endocrinology and Metabolism of Zhongshan Hospital, Fudan University, Shanghai, 200032 China; 2 Department of Immunology, Shanghai Medical College, Fudan University, Shanghai, 200032 China

**Keywords:** Differentiation, Obesity

## Abstract

Adipose tissue development begins in the fetal period, and continues to expand after birth. Dysregulation of adipose tissue during weaning may predispose individuals to lifelong metabolic disorders. However, the developmental remodeling of adipose tissue during weaning remains largely unexplored. Here we comprehensively compare the changes in mouse subcutaneous white adipose tissue from 7 days after birth to 7 days after weaning using single-cell RNA sequencing along with other molecular and histologic assays. We characterize the developmental trajectory of preadipocytes and indicate the commitment of preadipocytes with beige potential during weaning. Meanwhile, we find immune cells unique to weaning period, whose expression of extracellular matrix proteins implies potential regulation on preadipocyte. Finally, the strongest cell-cell interaction during weaning determined by the TGFβ ligand-receptor pairs is between preadipocytes and endotheliocytes. Our results provide a detailed and unbiased cellular landscape and offer insights into the potential regulation of adipose tissue remodeling during weaning.

## Abstract (web summary)

A single-cell sequence on mouse subcutaneous white adipose tissue analyses the dynamics of preadipocytes and immune cells populations in early life time and suggests potential changes involved in the beige-like remodeling during weaning.

## Introduction

White adipose tissue (WAT) is critical for maintaining energy homeostasis. White adipose development begins in the fetal stage, and it continues to expand after birth. Studies of human cohorts show that children and adolescents with obesity have a higher risk of developing metabolic disorders in adulthood^1–3^, suggesting that the normal development of adipose tissue in early life is crucial to metabolic homeostasis in adulthood. Human and experimental animal studies show that maternal nutrient challenges and stress during pregnancy and the postnatal period influence fat mass and leptin levels and even affect vulnerability to a high-fat diet later in life^4–6^, which also indicates that environmental insults during the critical period of developmental plasticity can elicit lifelong disabilities.

In addition to the perinatal period, weaning time seems to be another crucial period. Early in 1979, a study was performed to determine the adipocyte size in obese and nonobese human subjects ranging in age from 4 months to 19 years. The results showed that shortly after 1 year of age, the individual adipocyte size had decreased in the nonobese group, indicating a process of remodeling. However, adipocytes in the obese group continued increasing in size^7^. Notably, this important time point is around the weaning stage. Thus, remodeling during weaning seems to be crucial for body weight gain in later life. Observations in rodents also show high remodeling during weaning, as evidenced by the characteristic browning feature of subcutaneous adipose tissue^8–10^. We previously found that mice with adipose-specific knockout of BMPR2 died during weaning, although they appeared normal in the first two postnatal weeks^11^. Therefore, evidence in both humans and rodents supports that weaning time is a critical period when genetic and environmental influences can have lifelong effects.

WAT contains heterogenic cells, including adipocytes, immune cells, endotheliocytes, nerves, and progenitors. These cells are plastic and responsible for remodeling not only during development but also throughout adulthood for adaptation to environmental cues such as nutrition and temperature alterations^12^. Adipogenic progenitors give rise to functional mature adipocytes. Adipocytes with special functions, e.g., brown adipose activity, can be derived from specific progenitors^13,14^. A large number of studies have been carried out to determine what defines progenitors. Researchers have identified some molecules as markers of progenitors, such as *CD29*^15^, *DPP4*^16–18^, *Znf423*^19^, and *Pdgfra/b*^20–23^. Nonetheless, it seems difficult to identify the exact developmental stage and trajectory based on these data because these markers are studied separately by individual groups. More studies of comprehensive comparisons among those progenitor markers are needed.

Immune cells account for a great proportion of cells in WAT. A growing body of work is revealing the important role of immune cells, mostly focusing on how macrophages and T cells regulate metabolic homeostasis and disorders of adipose tissue, such as obesity^24,25^. Other studies have shown that type 2 immune responses involving type 2 innate lymphoid cells (ILC2s) and M2 macrophages regulate the beiging of WAT^26,27^. Considering the crosstalk between immune cells and adipocytes, immune cells undoubtedly contribute to adipocyte development and function. However, there have been few studies focusing on how immune cells are remodeled in developing adipose tissue.

Bone morphogenic protein (BMP) signaling plays very important roles in adipose tissue development^12^. With respect to the role of BMP in mesodermal formation^28^, BMP signaling definitely regulates the development of adipose tissue during the embryonic stage. We and other researchers have found that BMP signaling also regulates the metabolism of WAT in adulthood^29–32^. Our analysis of the *Bmpr2* knockout mouse phenotype revealed that *Bmpr2* knockout rendered adipocytes susceptible to inflammatory factor-induced death during weaning^11^, which indicates the important role of BMP signaling in normal remodeling in this period. BMPs are secreted peptides and serve as ligands that bind to type I and type II receptors on the cell surface and then activate the downstream p38/MAPK or Smad pathways. Since there are >30 secreted ligands, seven type I receptors, and five type II receptors, the binding of BMP ligands and receptors may link the communications between multiple types of cells in WAT^33–35^.

Considering the potential remodeling of WAT in weaning and its important role in metabolic homeostasis, in the present study, we comprehensively compared the changes in adipose tissue from 1 week (7 d) to 4 weeks (28 d) postnatally, including histological phenotyping and gene expression analysis using histologic methods and RNA sequencing. Moreover, to analyze the different cell groups and how their amount and function vary along with developmental time, we performed single-cell RNA sequencing. Basically, the metabolic activity is higher at the weaning stage than at an earlier time point (postnatal day 7); however, some immunoreactive processes, such as those involving NKT and T helper cells, are lower than those at a later time (postnatal day 28). We also found a cluster of preadipocytes with the potential to differentiate into thermogenic adipocytes during weaning. T cells and B cells specific to weaning characteristically express extracellular matrix (ECM). We further anticipated potential cell-cell communication based on the expression of ligands and receptors and found that the closest interaction was between preadipocytes and endotheliocytes, which was most prominent at weaning if determined by *Bmps*/*Tgfbs* and receptors. These results help to comprehensively elucidate the multiple biological processes occurring in the different types of cells during early life, especially weaning, and may attract attention to the crucially important period for intervening in metabolic disorders.

## Results

### Subcutaneous adipose tissue is remodeled at the weaning stage

**Fig. 1:**
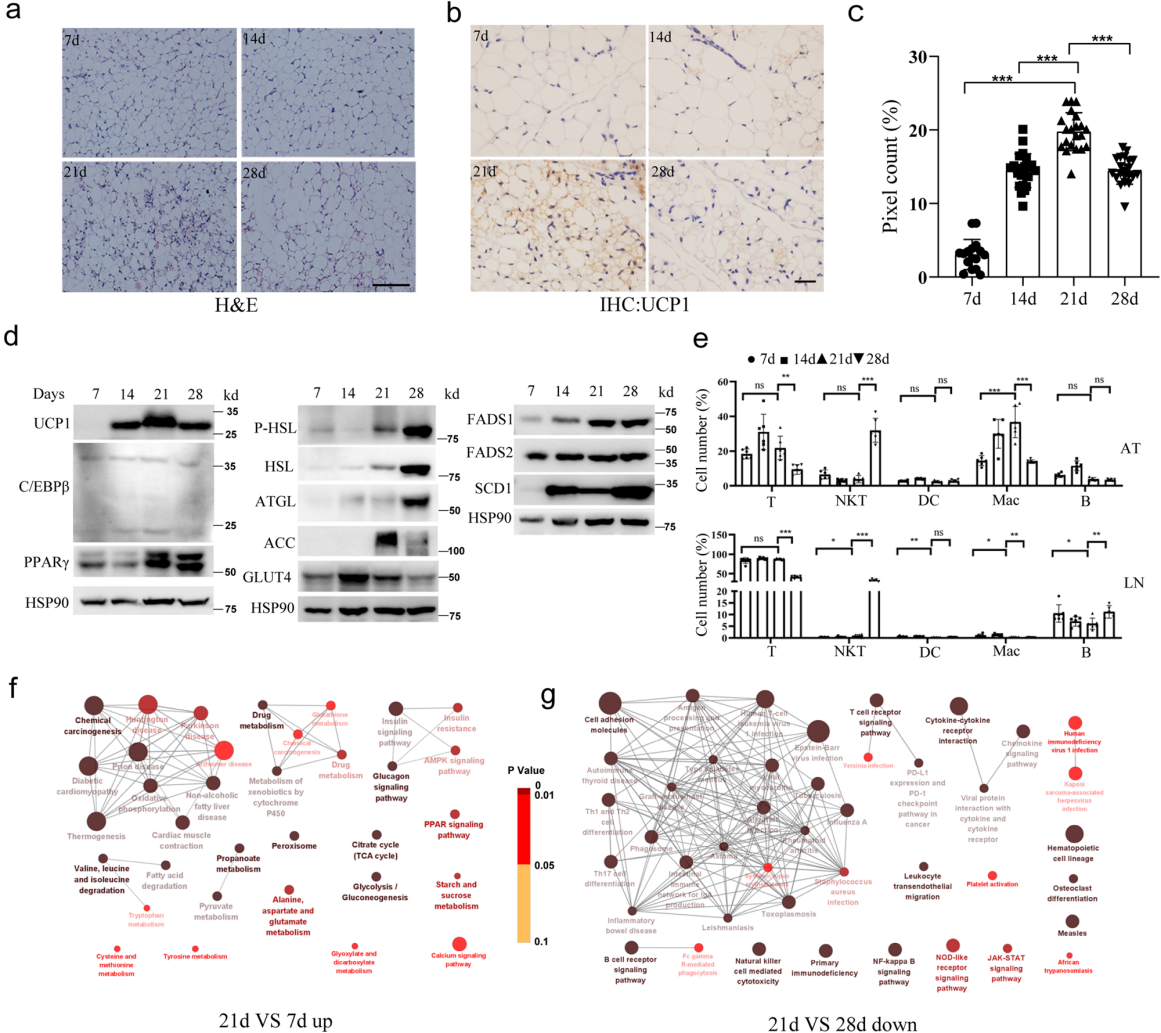
Phenotype and gene expression of mouse iWAT in early life. **a** H&E of iWAT collected from mice at different postnatal days. Scale bar, 100 μm. **b** IHC staining with anti-UCP1 antibody of iWAT. Scale bar, 20 μm. **c** Calculation and statics of UCP1 positive staining in **b** using image J with IHC plugin. Fields were from at least 3 individuals in each group with total number of 16–23. **d** Representative images of western blot of molecules expressed in iWAT at different postnatal days. **e** Cell number percentages of immune cells in CD45+ cells in iWAT excluding lymph node (LN) or LN by flowcytometry. **f**, **g** RNA-sequence of iWAT and differential expressed genes (|log2FoldChange|>1) were analyzed by KEGG and ClueGo to show the pathways (*p* value ≤ 0.05) and potential connections. Bar and error bar in **b** and **e** are mean and SD respectively. *P < 0.05, **P < 0.01, ***P < 0.005.

The death of *Bmpr2* knockout mice during preweaning indicates particular remodeling during this period^11^. Therefore, we first observed the histological changes in subcutaneous (inguinal) adipose tissue (iWAT) from postnatal day 7 (7 d) to day 28 (28 d). Postnatal day 21 (21 d) is the weaning point. We designated postnatal day 7 as infancy and postnatal day 28 as the juvenile stage according to physiological characteristics and sexual maturity. At 7 d, adipocytes exhibited typical morphological characteristics with one large lipid droplet filled, and then both the size of the cell and the lipid droplet increased at 14 days (14 d). However, at 21 d, adipocytes became more heterogeneous, with many multidroplet cells formed, which decreased at 28 d (Fig. 1a and Supplementary Fig. S1a). As adipocytes exhibited a multidroplet appearance, which is a phenotype of beige adipocytes, we then examined the expression of uncoupling protein 1 (UCP1), a protein that is indicative of thermogenesis. The immunohistochemistry (IHC) results showed a higher expression level of UCP1 at postnatal day 21. There was also some expression of UCP1 at 14 d and 28 d, but these levels were lower than those at 21 d. UCP1 was hardly detected at 7 d (Fig. 1b, c). The dynamic expression of UCP1 during development was further confirmed by western blotting (Fig. 1d). We further examined the expression of C/EBPβ (CCAAT/enhancer-binding protein beta), the key transcriptional regulator of adipogenic differentiation. The levels did not differ among the 4 time points, suggesting that adipocytes had already achieved adipogenic potential at 7 d postnatally. PPARγ (peroxisome proliferator-activated receptor gamma) is another key transcription factor for adipogenic differentiation. PPARγ was expressed at all four time points but was higher at and after 21 d of age. Because PPARγ also participates in other processes, such as mitochondrial biogenesis and lipid metabolism, the high level of PPARγ indicates active lipid metabolism and mitochondrial function. The expression of the lipases responsible for lipolysis, that is, adipose triglyceride lipase (ATGL) and phosphorylated and nonphosphorylated hormone sensitive lipase (HSL), gradually increased over the course of development time, reaching the highest level at 4 weeks. Acetyl-CoA carboxylase (ACC) is the key enzyme for fatty acid synthesis, and its expression level was highest at 21 d. Glucose transporter 4 (GLUT4) was most highly expressed at 14 d. As the degree of fatty acid saturation is related to the function of lipids, we determined the levels of three desaturating enzymes. Fatty acid desaturase 1 (FASD1) increased from early to later time points, while FADS2 did not change. The level of stearoyl-CoA desaturase 1 (SCD1) was extremely low at 7 d and was high at and after 14 d (Fig. 1d).

In addition to the main histological and molecular manifestations mostly related to adipocytes, we also examined the number of immune cells (Fig. 1e and Supplementary Fig. S1b). As the iWAT has an embedded lymph node (LN), we examined and calculated the cells from these two regions separately. There are many T cells and B cells in LNs. Among the CD45-positive immunocytes, the percentage of T cells was lowest at 28 d, but NKT cells, defined by NK1.1, were extremely highly abundant at this time point. The percentage of B cells in LNs was lowest at 21 d. Dendritic cells (DCs) and F4/80-positive macrophages (Macs) occupied a very small percentage in LNs, and their percentages at 21 d were lowest among their respective cell types. Adipose tissue (AT), excluding LNs, also has immune cells. There was a higher percentage of T cells at 21 d than at 28 d; however, the percentage of NKT cells was extremely high at 28 d. B cells did not change much at 7 d, 21 d, and 28 d, while the highest percentage of macrophages was found at 21 d.

The above data revealed the dynamics of metabolism and immunoactivity along with development during early life based on the expression of selected marker genes. To examine the alterations from a comprehensive perspective, we performed bulk RNA sequencing of iWAT at the four time points. After comparing the differentially expressed genes and performing KEGG analysis, we found that the expression of the genes responsible for energy metabolism, such as mitochondrial function, TCA cycle, insulin signaling and PPAR signaling, increased from 7 d (infancy period) to 21 d (weaning period) (Fig. 1f and Supplementary Fig. S1c), while the expression of genes associated with ECM action decreased (Supplementary Fig. S1e, f). Compared to the juvenile period (28 d), the weaning period (21 d) had lower levels of some immune reactions, such as T-cell differentiation and natural killer cell-mediated cytotoxicity (Fig. 1g and Supplementary Fig. S1d), but higher activity of drug metabolism by cytochrome P450 (Supplementary Fig. S1g, h).

### Single-cell RNA-seq reveals iWAT cell heterogeneity and dynamics in early life

**Fig. 2:**
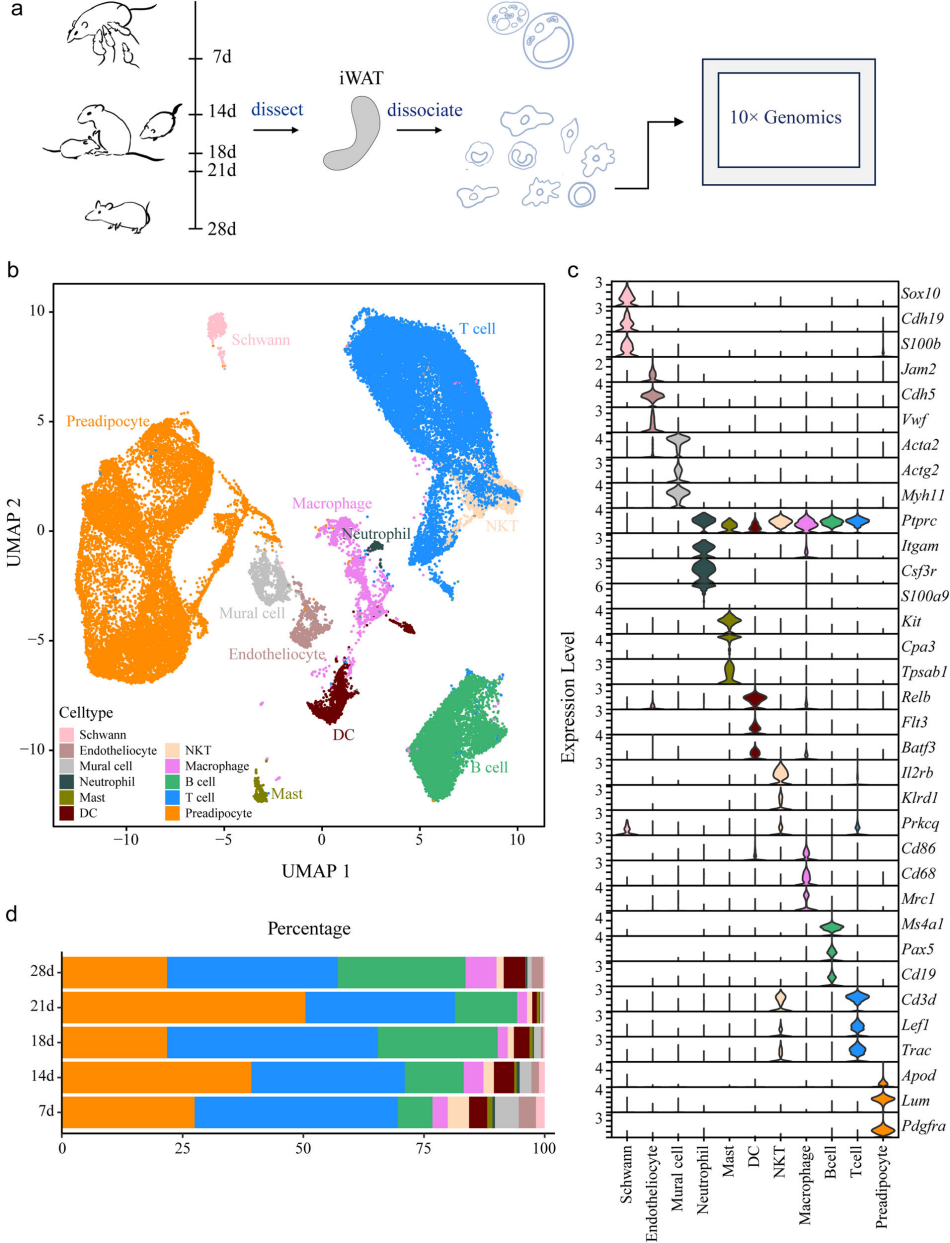
Cell type annotation of SVF cells in mouse iWAT. **a** Schematic workflow of Sc-RNA sequencing of iWAT. **b** Uniform manifold approximation and projection (UMAP) projection of clusters formed by 48,668 mouse stromal vascular cells in iWAT. **c** Expression of marker genes for each cell types. **d** Cell number percentages of different cell types.

Considering the apparent alterations in phenotype and gene expression in mouse iWAT during early life, we further performed single-cell RNA sequencing (scRNA-seq) to profile cells in an unbiased manner from iWAT. We collected iWAT from 7-, 14-, 18-, 21-, and 28-day-old mice and enzymatically disassociated the tissues into single cells. Stromal vascular cell fractions (SVFs) were separated from mature lipid-containing adipocytes by centrifugation and were subjected to scRNA-seq (Fig. 2a). We performed nonlinear reduction by uniform manifold approximation and projection (UMAP) and unsupervised clustering on the basis of transcript levels. The clusters were annotated and classified into 11 cell types, including preadipocytes, T cells, B cells, macrophages, natural killer T cells (NKT cells), mural cells, endotheliocytes, schwann cells, dendritic cells (DCs), mast cells, and neutrophils (Fig. 2b). Cell type annotation was based on well-established markers, and representative differential expressed genes (DEGs) of each class are shown in Fig. 2c. Analysis of the frequency of cells in each class (Fig. 2d and Supplementary Fig. S2a) revealed that preadipocytes occupied a large percentage of all cells and that T cells were comparable to preadipocytes. The percentage of preadipocytes reached the highest level during the weaning period (21 d); however, the percentages of macrophages, NKT cells and DCs were lowest at 21 d. The primary analysis of those heterogenic cell types showed that they are not constant in early life, indicating active remodeling of adipose tissue.

### Clustering and reconstruction of the developmental trajectory of preadipocytes

**Fig. 3:**
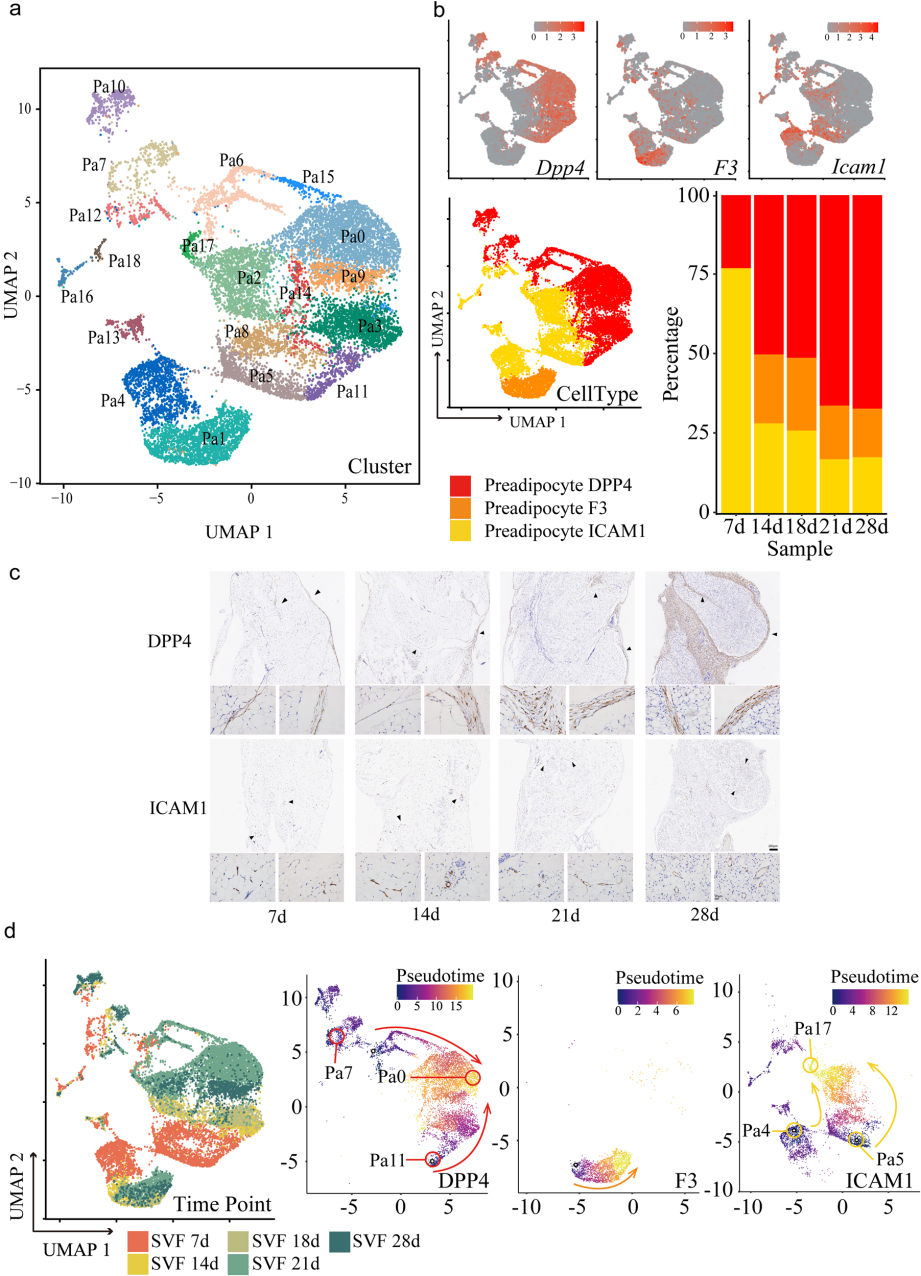
Clustering and developmental trajectory of preadipocytes. **a** UMAP projection of cluster Pa0-Pa18 formed by 1,6497 preadipocytes. **b** UMAP projection of clusters showing DPP4, ICAM1 and F3 expression, and cell number percentages of the three populations. **c** IHC of iWAT with DPP4 and ICAM1 antibody. Scale bar 200μm for zoom out, 20μ for zoom in. **d** UMAP projection of pradipocytes clusters with timepoints information.

To more closely inspect the preadipocytes, we reclustered preadipocytes into 19 clusters designated Pa0-Pa18 (Fig. 3a) according to characteristic gene expression (Supplementary Fig. S3a and Supplementary Data 1). Cell counts decreased from Pa0 to Pa18 (Supplementary Fig. S3b). Gene accounts were high in Pa17 and Pa18 (Supplementary Fig. S3b).

Merrick D et al. profiled progenitor cells from the developing iWAT of 12-day-old mice by scRNA-seq and identified a developmental hierarchy from DPP4 (dipeptidyl peptidase–4)-expressing multipotent cells to either ICAM1 (intercellular adhesion molecule–1)-expressing committed preadipocytes or an adipogenic cell population marked by F3/CD142 expression^18^. In the present study, we collectively designated all clusters of preadipocytes as DPP4, F3 and ICAM1 preadipocytes according to their expression (Fig. 3b, Supplementary Fig. S3c). We verified the expression of some genes reported in Merrick D’s study, including *Akr1c18*, *Cd55*, and *Anxa3* in DPP4 subpopulation, as well as *Vcam1*, *Fabp4*, and *Ggt5* in ICAM1 subpopulation. In addition, we found some more genes enriched in each subpopulation, such as *Wnt2* and *Efhd1* expressed in DPP4 subpopulation, *Mgp*, *Mmp3*, and *Fmo2* in F3 subpopulation, and *Adam12* and *Prlr* in the ICAM1 subpopulation. To find out the dynamics of preadipocytes associated with weaning, we observed their alteration along the course of developmental time. We firstly examine the distributions of DPP4 and ICAM1 positive cells by staining with IHC method. DPP4 positive cells located in connective tissues outlining the whole adipose tissue or the lobes within. ICAM1 positive cells located in vascular structures that were more likely capillary at 7d and became typical tube-like at 28d (Fig. 3c). It was hard to quantitate the density of positive cells because they were not evenly distributed. Analysis of Sc-sequencing data showed iWAT from 21d- and 28 d-old mice had abundant DPP4 preadipocytes; however, that from 7-d-old mice had the highest percentage of ICAM1 preadipocytes (Fig. 3b). F3 preadipocytes did not appear at 7 d, and the percentage did not change much at 14 d and later (Fig. 3b). It seemed that the hierarchy defined by the three marker genes did not align with developmental time. Therefore, we integrated our data and Merrick D’s data to generate the landscape of mice preadipocyte development including seven timepoints 7 d, 12 d, 14 d, 18 d, 21 d, 28 d, and adult). We observed that cells of the two datasets were matched well, in particular for the preadipocytes (Supplementary Fig. S3d). We also focused on preadipocytes for further investigation and verified that the 12 d preadipocytes in Merrick D’s data have a close relationship with our 7 d and 14 d preadipocytes. While the adult cells in Merrick D’s data were located near the group of 28 d from our data. Once we set 7 d cells as the root for trajectory analysis and observed the same differentiation direction as our previous versions.

We then marked all groups of preadipocytes by timepoint and tried to reconstruct the developmental trajectory (Fig. 3d). Overall, both DDP4 and ICAM1 groups began at 7d and ended at 21d, indicating the process of adipogenic commitment at weaning. DPP4 preadipocytes began with Pa7 or Pa11 at 7 d and ended with Pa0 at 21 d. Surprisingly, KEGG and GO analyses found that Pa7 expressed genes for T-cell differentiation and cell-cell adhesion (Supplementary Fig. S4a, b). Pa11 responded to BMPs and TGFβ, and the serine/threonine kinase pathway was activated (Supplementary Fig. S4a, b). Genes enriched in Pa0 were involved in WAT development, such as the WNT signaling pathway and fatty acid metabolism (Supplementary Fig. S4c, d). The percentage of Pa0 in all preadipocytes was approximately 40% at 21 d (Supplementary Fig. S4e), which was most abundant than at any other time points. ICAM1 preadipocytes began with Pa4 and Pa5 at 7 d and ended with Pa17 at 21 d (Fig. 3c). KEGG (Supplementary Fig. S5a) and GO (Supplementary Fig. S5b) analyses showed that the pathways that were active in Pa4 mainly involved inflammatory reactions, while those in Pa17 were more involved in mitochondrial function. Moreover, Pa17 cells expressed *Cebpa* (CCAAT/enhancer-binding protein alpha), *Pparg* and *Cebpb* for adipocyte differentiation, as well as adiponectin, *Plin1* (perilipin 1), *Lipe* (lipase E), *Acsl1* (acyl-CoA synthetase long chain family member 1), *Dgat2* (diacylglycerol O-acyltransferase 2) and *Agpat2* (1-acylglycerol-3-phosphate O-acyltransferase 2) for lipid formation (Supplementary Fig. S5c). They also expressed *Ppargc1b* (PPARG coactivator 1 beta) (Supplementary Fig. S5c), which is expressed in mature adipocyte as well as preadipocyte^15^. From the data on Pa0 and Pa17, we predicted that the two subsets recruited at 21 d are closer to adipocytes, re-enforcing the process of commitment and further differentiation at weaning.

Expression of F3 was restricted in Pa1 and Pa4. Cells in this class seemed to develop from Pa4 to Pa1 if aligned with the developmental process. Genes enriched in Pa1 participate in complement activation and EMC construction (Supplementary Fig. S6a, b).

### Identification of active preadipocytes during the weaning period

**Fig. 4:**
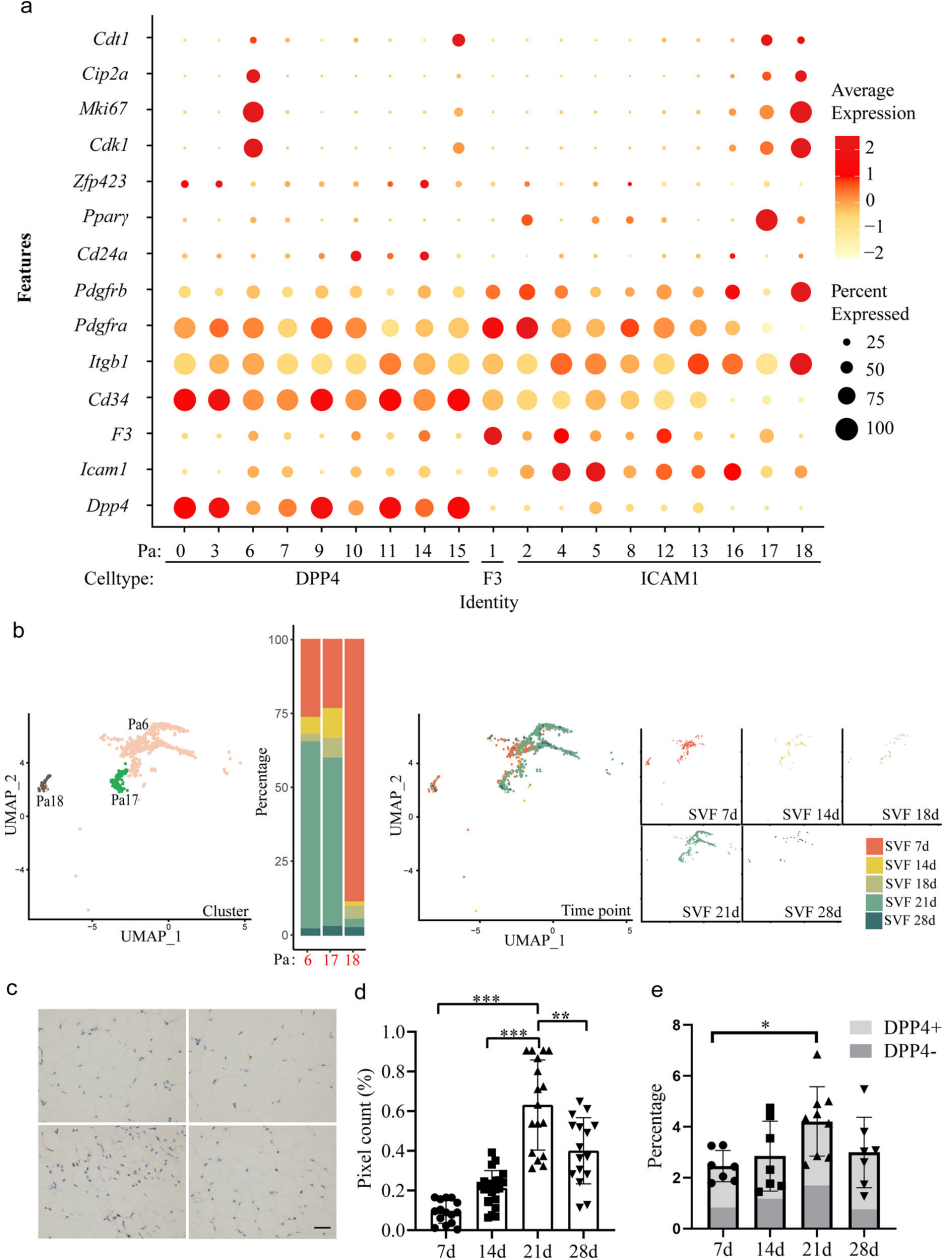
Identification of active preadipocytes during weaning time. **a** Expression of reported preadipocyte markers in all Pa0-Pa18 clusters. **b** UMAP projection of clusters 6, 7 and 18, and cell percentage of the three cluster. **c** IHC of Mki67 showing the proliferating cells Scale bar: 20 μm. **d** Positive staining was calculated using ImageJ with IHC plugin. Fields were from at least 3 individuals in each group with total numbers of 15–21. **e** Cell number percentages marked with Cip2a, DPP4 in CD45 negative SVF cells detected by flowcytometry. *n* = 7 in 7d, 14d, and 28d group. *n* = 9 in 21d group. Bar and error bar in **d** and **e** are mean and SD respectively. *P < 0.05, **P < 0.01, ***P < 0.005.

We further examined the expression of the reported progenitor markers in 19 preadipocyte groups (Fig. 4a). *Cd29* (integrin subunit beta1, *Itgb1*) unanimously appeared in most cells in all groups, with the strongest expression in Pa18, while *Cd34* was expressed in almost all groups except Pa16, Pa17 and Pa18. Very low percentages of preadipocytes expressed *Cd24a*, as well as *Zfp423*. *Pparγ* expression was mostly restricted to Pa17. *Pdgfrα* (platelet-derived growth factor receptor alpha) expression was distributed in all clusters except Pa18. *Pdgfrβ* expression was extremely high in Pa18. It is very intriguing that Pa6, and Pa18 had high proliferative activity as they highly expressed the cell cycle-related genes *Mki67* and *Cdk1*. Pa16 also possessed proliferative potential as it expressed some levels of *Cdt1*, *Mki67* and *Cdk1*. Pa6 and Pa17 were abundant at 21d, while Pa 18 enriched at 7d (Fig. 4b). IHC of *Mki67* in iWAT tissues gave rise to the highest level of positive staining at 21 d (Fig. 4c, d). We then calculated proliferating cells by flow cytometry. We used *Cip2a* because it is located in the cell membrane and positively regulates cell proliferation. *Cip2a*-positive cells in the *Cd45*-negative region increased at 21 d, among which both *Dpp4*-positive and *Dpp4*-negative cells contributed (Fig. 4e). Further KEGG analysis of Pa6 verified its proliferative capacity because most of the top 15 significant pathways were related to the cell cycle (Supplementary Fig. S7a). Pa18 also expressed genes related to the cell cycle; however, there were many genes involved in pathways related to mitochondrial function, such as Parkinson’s disease, prion disease, oxidative phosphorylation, and thermogenesis (Supplementary Fig. S7a), indicating that the descendant adipocytes of the two groups may have different functions.

### Characterization of immune cells and their link to adipose tissue development

**Fig. 5:**
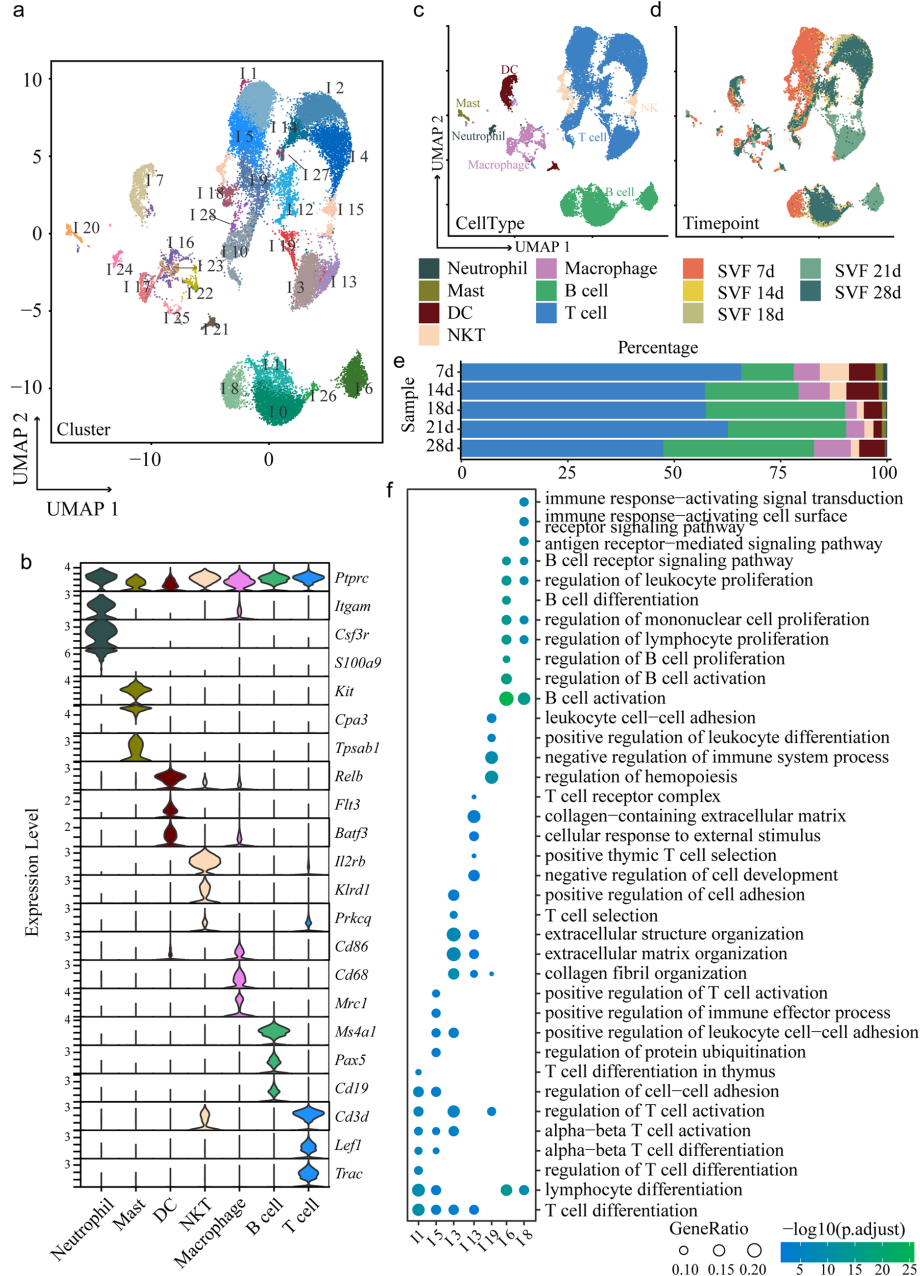
Clusters of immune cells and their link to adipose tissue development. **a** UMAP projection of cluster I0-I28 formed by 2,9493 immune cells. **b** Expression of marker genes for each cell type. **c** UMAP projection of clusters with cell type annotation. **d** UMAP projection of clusters with timepoint information. **e** Cell number percentages of all different immune cell types. **f** GO analysis of I1,5, 8 and I3,13,19,6.

The function of immune cells in adipose tissue has attracted the attention of researchers. Reports have mainly focused on T cells and macrophages. To obtain a more comprehensive view of immune cells in iWAT and their dynamics during development, we reanalyzed all immune cells. We re-clustered immune cells into 29 clusters named I0-I28 (Fig. 5a) according to characteristic gene expression (Supplementary Fig. S8a and Supplementary Data 2), and representative marker genes of each cluster are shown in Supplementary Fig. S8b. The clusters were annotated and classified into 7 cell groups, namely, neutrophils, mast cells, DCs, NKT cells, macrophages, B cells and T cells (Fig. 5b, c), and the compositions of subgroups over time were examined (Fig. 5c–e). Overall, approximately half of the immune cells were T cells, and B cells were the second most abundant cell type, followed by macrophages. The percentages of NKT cells and DCs were comparable to those of macrophages, while those of mast cells and neutrophils were very small. The GO and KEGG analysis verified that clusters of the same cell type have the same function, such as DEGs genes in I0, I6, I8, I11, and I26 from B cell are all show B cell activation function in GO analysis, and I0, I6, and I26 from B cell are all participate in B cell receptor signaling pathway in KEGG analysis (Supplementary Fig. S8c).

After examining how those immune cells were distributed at different developmental timepoints (Fig. 5a, c, d), we found that the types of immune cells clustered at 14 d, 18 d, and 28 d were similar. However, the clustering at 7 d and 21 d had individual characteristics. We then found that two subclusters of B cells, namely, I6 and I8, were timepoint specific (Fig. 5a, d and Supplementary Fig. S8d). I6 B cells appeared only at 21 d, and I8 appeared at 7 d. It was expected that GO analysis of genes in the two clusters would be mostly relevant to B-cell proliferation and activation (Fig. 5f), but after comparing the pathways generated by KEGG analysis (Supplementary Fig. S8e), we found pathways unique to individual classes. Pathways active only in the I6 B cell cluster mainly resulted from the expression of some types of collagens, and pathways specific for I8 involved genes regulating cell cycle progression and cell death, such as apoptosis and ferroptosis.

Similar to B cells, there were also T cell classes appearing only at 7 d or 21 d. I1 and I5 were present at 7 d, and I3, I13, and I19 were present at 21 d. GO analysis showed that I1 cells were the T cells underlying differentiation and activation, and I5 T cells responded to IL-4 (Fig. 5f), indicating that they might be developing into Th cells. I3, I13 and I19 T cells at 21 d highly expressed proteins for ECM organization, such as collagens and fibronectin (Fig. 5f, Supplementary Fig. S8f), which was similar to the B cell protein expression at this timepoint.

### Cell-cell interaction in iWAT determined by the TGFβ superfamily

**Fig. 6:**
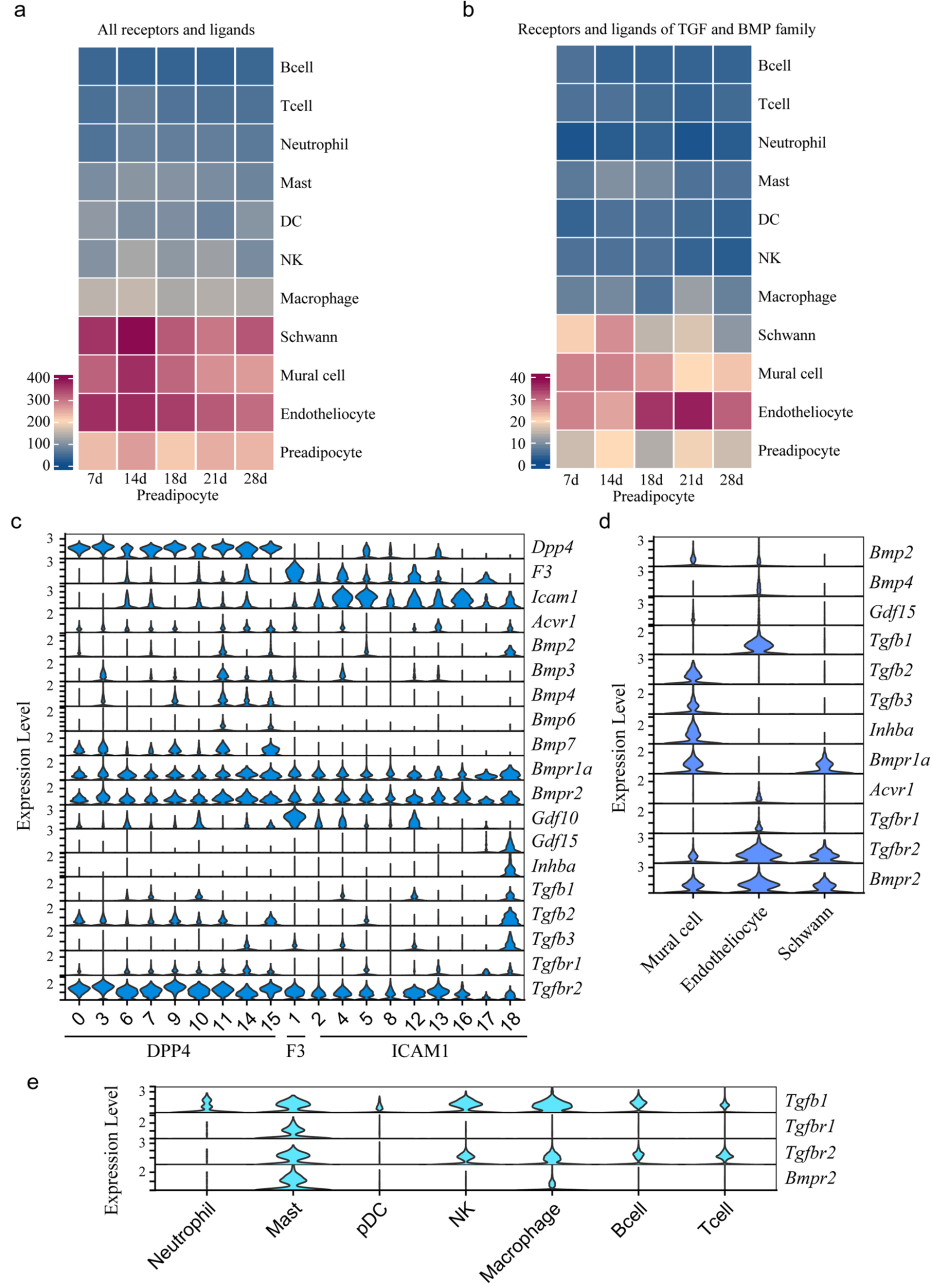
Cell-cell interaction in iWAT determined by ligand-receptors. **a** Heat map showing number of significant interactions between preadipocyte with other cell types in iWAT at different time points as determined by CellphoneDB with all receptors and ligands. **b** Heat map showing number of significant interactions between preadipocyte with other cell types in iWAT at different time points as determined by CellphoneDB with all receptors and ligands of TGFβ superfamily. **c** Expression of receptors and ligands of TGFβ superfamily in Pa0-Pa18 preadipocyte. **d** Expression of receptors and ligands of TGFβ superfamily in mural cells, endothyliocyte and schwann cell. **e** Expression of receptors and ligands of TGFβ superfamily in immune cell groups.

Since both development and remodeling involve the coordination of all cell types, potential intercellular communication would provide clues for deciphering biological programs in either physiologic or pathologic contexts. We used CellPhoneDB, which uses the expression of ligand-receptor pairs to indicate cell-cell interactions^36^. Unselected ligand and receptor pairs indicated that preadipocytes most robustly communicated with endotheliocytes, followed by mural cells, schwann cells, and macrophages. Preadipocytes also communicated extensively with mast cells at 14 d, although less so at other times (Fig. 6a). After calculating all ligand-receptor pairs across all families, we observed that BMP family was ranked top No.2, just after the communication of adhesion created by collagens and integrin, which are universal for cell-cell communications (Supplementary Fig. S9a).

BMP signaling has been demonstrated to play an important role in the development and metabolism of adipose tissue. BMPs belong to the TGFβ superfamily. The ligands function by binding with receptor tetramers containing both type 1 and type 2 receptors. We previously found that *Bmpr2* knockout mice hardly survived through weaning due to a deficiency of WAT, indicating that *Bmpr2* signaling is crucial for the remodeling of WAT during periweaning. We then used ligand‒receptor pairs of the BMP family as well as other members of the TGFβ superfamily to analyze how much preadipocytes communicate with other cell types (Fig. 6b). The results showed that preadipocytes had the strongest interaction with endotheliocytes, especially at 21 d. Preadipocytes also communicate extensively with mural cells and Schwann cells. The connection with mural cells was relatively weak at 21 d, while the connection with Schwann cells was relatively strong at 14 d. Notably, although interactions between preadipocytes and endotheliocytes determined by the TGFβ superfamily were relatively high at 21 d, those determined by all ligands and receptors were low at 21 d when compared with other timepoints, indicating that BMP and TGFβ members are extremely important during the weaning period.

To further determine how different ligands and receptors function in specific cell classes, we examined the expression and distribution of all reported members of the TGFβ superfamily in preadipocyte clusters, and detectable expression is shown in Fig. 6c. *Bmpr2* is expressed in all classes of preadipocytes. Moreover, *Bmpr1a* and *Tgfbr2* were expressed in all preadipocytes. DPP4 preadipocytes also expressed *Tgfbr1* and *Acvrl* to some extent. Regarding ligands, DPP4 preadipocytes expressed *Bmp7* and *Tgfb2*. *Gdf10* was mostly enriched in F3. It was intriguing that Pa18, the class with proliferative potential and *Pdgfrβ* expression (Fig. 4a), had more types of ligands than other preadipocytes, including *Bmp2*, *Gdf15*, *Inhba*, *Tgfb1*, *Tgfb2*, and *Tgfb3*. The *Tgfb* ligands in Pa18 might imply its potential for inflammatory actions. Endotheliocytes, which communicated most with preadipocytes, highly expressed *Bmpr2*, *Tgfbr2* and *Tgfb1* (Fig. 6d). By matching ligands and receptors, DPP4 preadipocytes and Pa18 ICAM1 preadipocytes may actively interact with endotheliocytes. Mural cells and Schwann cells expressed *Bmpr2*, *Bmpr1a*, and *Tgfbr2*, which might account for their good connection with preadipocytes. Mural cells also expressed ligands such as *Tgfb2* and *Tgfb3*, whose receptors are *Acvrl* or *Tgfr1* among type 1 receptors and *Tgfr2* among type 2 receptors. Another ligand, *Inhba*, synthesized by mural cells bound to *Acvr1* and *Acvr2*, which were expressed at detectable levels in DPP4 preadipocytes (Fig. 6d).

*Tgfb1* secreted by immune cells might contribute to the communication with preadipocytes and was expressed mainly in macrophages, NKT cells and mast cells (Fig. 6e). *Tgfb1* was reported to inhibit the progression of DPP4 progenitors into a more adipogenic state^18^, which was feasible based on our observation of *Tgfbr2* expression in DPP4 preadipocytes (Fig. 6c). In addition to the *Tgfb1* ligand, mast cells showed high expression of *Tgfbr1*, *Tgfbr2*, and *Bmpr2* (Fig. 6e), suggesting communication with preadipocytes that had not yet been noted.

## Discussion

In this study, we proposed that weaning is a particular period in early life when iWAT undergoes remodeling that might have long-term effects in adulthood. The remodeling was evidenced by alterations in the expression of genes involved in thermogenesis and lipid and glucose metabolism. In addition to metabolic genes, genes for immune regulation were also greatly changed, as exhibited by the bulk RNA sequencing of iWAT. We then obtained a more comprehensive atlas of mouse iWAT across the developmental stages in early life by utilizing powerful single-cell RNA sequencing. Our analysis reveals some preadipocyte and immune cell subpopulations that are unique to weaning time, as well as potential communication links between preadipocytes and other cell types. These findings provide a comprehensive resource for understanding the heterogeneity of preadipocyte contributing the phenotype and function of adipose tissue for future exploring the mechanism of regulation of adipose tissue development that is vital to metabolism.

The adipose tissue at weaning display high *Ucp1* level. we have been trying to figure out what kind of preadipocytes accounting for high level of *Ucp1* after forming adipocytes. As preadipocytes did not express *Ucp1*, we supposed those preadipocytes with high level mitochondrial activity have potential to differentiated into adipocytes with high level of *Ucp1*. By KEGG and GO analyzing of clusters, we found Pa17 expressed high levels of mitochondrial genes. As Pa17 seemed to be more adipogenic committed (Supplementary Fig. S5b), the up-regulated phosphorylation (OXPHOS) and mitochondria-related genes may be resulted from later adipogenic stage based on the observation by other researchers. Nevertheless, Pa17 still maintained the proliferation potential and extremely high level of *Pparg*-the gene expressed in some kind of preadipocytes^37^. So, the possibility that Pa17 is a precursor with beige fate could not be excluded. Pa18 maintained high expression level of genes for both cell cycle and complex of ETC of mitochondrial. Meanwhile, Pa18 specifically expressed *Pdgfrb*, which was traced by other researchers to contribute to beige adipocyte in cold condition^23^. However, Pa18 enriched at 7d but not at weaning time, so the group of preadipocyte might not be activated by weaning. It was noted that Pa0 cluster DPP4, which enriched t at 21d and reached to the percentage of about 40% in total number of SVF. The GO analysis revealed the active function for fatty acid metabolism and angiogenesis regulation, also suggesting its potential to lead to beige-like phenotype. Collectively, Pa17 and Pa0 identified at 21d might account for the beige-like phenotype and high level of UCP1 at weaning time.

A previous study defined the developmental hierarchy of DPP4+ progenitors that give rise to committed ICAM1+ and CD142+ preadipocytes, which are prone to differentiate into mature adipocytes^18^. We found that DPP4- and ICAM1-positive cells both formed at 7 d and existed at all timepoints observed. If aligned with the developmental time, DPP4 and ICAM1 both have a trajectory from 7 d to 21 d. At 21 d, the DPP4-positive subcluster Pa0 expressed the multipotent stem cell markers *Creb5* and *Wnt*, while the ICAM1 subcluster at this time expressed genes for committed adipogenic potential, such as *Cebpa*, *Pparg* and *Cebpb*. From this point of view, DPP4 cells are upstream of the developmental hierarchy. However, from the in situ distribution of DPP4 and ICAM1 positive cells, DPP4 and ICAM1 might identified two distinct preadipocyte derived from individual ancestor, which ultimately gave rise to the heterogeneity of adipocytes. KEGG analysis showed that both the DPP4 and ICAM1 subclasses at 7 d were involved in inflammatory reactions, among which many genes are responsible for chemotaxis or migration. This implies the possibility that some precursors develop into cells with inflammatory regulation potential, such as those defined by Gupta’s groups as fibro-inflammatory progenitors (FIPs)^38,39^. However, considering the function of these molecules, they might drive cells to migrate during the differentiation process, since stem cells are thought to localize in the reticular interstitium (RI) outlining adipose tissue or the vascular wall but eventually disperse in the whole tissue after they have differentiated into mature adipocytes. Recent studies about Wnt signaling in progenitors show those non-differentiated cells that maintain progenitor characteristics are Wnt pathway active and express gene sets such as external encapsuling structure organization and connective tissue development^40,41^, which seems match the structural localization and migration potential of DPP4 cells. Intriguingly, our data analysis also revealed coincidence of wnt2 and DPP4 (Supplementary Fig. S3c). So, those data along with other reports may provide resource for comparing adipogenic progenitor markers involving dpp4 and wnts.

Immune cells are major components of adipose tissue. Research on immune cells in adipose tissue has been expanding from inflammation during obesity development to the functions that regulate thermogenic fat and energy expenditure^24–27^. In either situation, T cells and macrophages are usually characterized by the secretion of cytokines to coordinately activate other immune cells or affect adipocytes directly. Findings about macrophages reveal more secretory factors, such as choline and catecholamines. We previously identified that slit3 activates sympathetic nerves in adipose tissue and stimulates thermogenesis^42^. Therefore, the functions of immune cells are far more numerous than we had recognized. It is intriguing that T-cell and B-cell subsets enriched at weaning characteristically express genes of the ECM, such as *Col6a1*, *Col6a3*, *Col5a1*, *Col5a2*, and *Fn1* (Supplementary Fig. S8f). While our analyses removed doublet and strictly controlled the ambient RNA, the possibility of T and B cell regulating development through ECM needs further exploration.

During weaning, the diet of juvenile mice gradually shifts from milk to the adult diet. Young mice are supposed to feed totally on milk at 7 d, on a mixed diet of both the mother’s milk and food at d 14 and d 18, to be weaned at d 21 and to have adapted to being an adult for a week at 28 d. Thus, remodeling may represent nutrient adaptation. However, the clustering of immune cells showed that the subclusters at d 14 and d 18 were rather similar to those at d 28 but different from those at d 21, which indicates that the cellular and histological properties of iWAT at 21 d may represent developmental processes other than the shift in diet.

## Methods

### Animals

All animal experiments were approved by the Fudan University Shanghai Medical College (No. 20180302-010). Mice were C57BL6/J background and housed under a 12 h light-dark cycle. Housing temperatures and humidity were kept within arrange of 21.7–22.8 °C and 50-60%, respectively. Mice of different ages were included and compared from the same litter. We have complied with all relevant ethical regulations for animal use.

### Single cell capture, library construction and sequencing

Adipose tissues for cell preparation were from the male mice of two litters. At each time point, two mice, one from litter #1 and the other from litter #2, were sacrificed and subcutaneous iWAT were dissected and pooled. The lymph nodes in the tissues were not intended removed. Cells of stromal vascular fraction (SVF) of iWAT were prepared by enzymatic dissociation with collagenase VIII (sigma c2139)^29^. After removing dead cells with the dead cell remove kit (Miltenyi. 130-090-101), live cells were loaded into Chromium microfluidic chips and barcoded within a 10× Chromium Controller (10X Genomics), Single cell transcriptome were reverse-transcribed into cDNA library containing 10X cell barcodes and unique molecular identifiers (UMI) information. All procedures were performed with reagents from Chromium Next GEM Single Cell 3ʹ GEM, Library & Gel Bead Kit v3.1 (10X Genomics, Cat. No. 1000121), following the manufacturers’ protocol. All the libraries were sequenced in a PE150 mode (Pair-End for 150 bp read) on the NovaSeq platform (Illumina).

### Single-cell sequencing data processing

Raw sequencing reads of the cDNA library were mapped to the reference genome by 10X Genomics Cell Ranger (version 3.1.0)^43^. Genome Reference Consortium Mouse Build 38 (GRCm38) was used as reference for 10X Cell Ranger. High fraction reads in cells, and characteristic steep cliff of barcode rank plots indicated the negligible ambient RNA. Doublets cells were filtered out by Scrublet (https://github.com/swolock/scrublet)^44^.

Seurat (version 4.0.5, https://satijalab.org/seurat/index.html) was used to present quality control and defined cell types for all samples^45^. Cells with more than 20% mitochondrial UMI, less than 200 genes or more than 6000 genes were excluded from the downstream analysis. Genes with the log-fold change≥0.25and FDR < 0.05 were considered as differentially regulated genes of white adipocyte tissue. Then 31,053 features for 46,684 cells were used, and calculating clusters using a Louvain algorithm, 15 principal components, and a resolution of 0.5. Clusters were identified as neutrophils, mast cells, dendritic cells, Schwann cells, endotheliocytes, mural cells, nature killer cells, macrophages, B cells, T cells and preadipocytes using marker genes identified by previous studies^46–51^. Marker *Csf3r*, *Itgam*, *S100a9* was used to define neutrophils; Marker *Cpa3*, *Kit*, *Tpsab1* was used to define mast cells; Marker *Flt3*, *Relb*, *Batf3* was used to define dendritic cells; Marker *Sox10*, *Cdh19*, *S100b* was used to define Schwann cells; Marker *Jam2*, *Cdh5*, *Vwf* was used to defined endotheliocytes; Marker *Acta2*, *Actg2*, *Myh11* was used to defined mural cells; Marker *Il2rb*, *Klrd1*, *Prkcq* was used to defined nature killer cells; Marker *Cd86*, *Cd68*, *Mrc1* was used to defined macrophages; Marker *Ms4a1*, *Pax5*, *Cd19* was used to defined B cells; Marker *CD3d*, *Lef1*, *Trac* was used to defined T cells; Marker *Pdgfra*, *Lum*, *Apod* was used to defined preadipocytes. The marker genes for each cell type (e.g., preadipocytes, T cells and B cells) were obtained from previous studies. For example, markers of preadipocytes were obtained from Emont M, and Bäckdahl J^52^. Markers of T cells and B cells were obtained from Emont M and CellMarker^47^.

We further integrate clusters of these 15,348 cells which were defined as preadipocyte into DPP4, F3 and ICAM1 by specific expressed genes followed previous studies. Cells specific expressed *Dpp4* and DPP4 subpopulation markers were used to defined as DPP4 preadipocyte; Cells specific expressed *F3* and F3 subpopulation markers were used to defined as F3 preadipocyte; Cells specific expressed *Icam1* and ICAM1 subpopulation markers were used to defined as ICAM1 preadipocyte. Furthermore, the markers of DPP4 subpopulation such as *Akr1c18, Cd55, Anxa3*, markers of ICAM1 subpopulation such as *Vcam1, Fabp4*, and *Ggt5* were obtained from Merrick D. We continue integrate clusters of these 29,493 cells which specific expressed Ptprc were defined as immune cells into previous classification. Seurat was also used to find all DEGs in subclusters, and we set cutoff of FDR > 0.05, foldchange > 1.5 and pct.1 > 0.3. In addition, we also used the function FindAllMarkers (parameter: only.pos = TRUE, min.pct = 0.25, thresh.use = 0.25) to screen specifically DEGs of each subpopulation across preadipocytes and immune cells. These DEGs set were used to investigate gene ontology (GO) and kyoto encyclopedia of genes and genomes (KEGG) analysis with R package clusterProfiler (version 4.3.0.991, http://bioconductor.org/packages/release/bioc/html/clusterProfiler.html)^53^. We provide all these genes in Supplementary Data 3–6.

In order to investigate the dynamic development along with time in preadipocytes. We use monocle3 (version 1.0.0, https://cole-trapnell-lab.github.io/monocle3/) to construct trajectories of DPP4, ICAM1 and F3 preadipocyte, and order the beginning of cells in pseudotime based on preadipocyte clusters from 7 day^54^.

To infer cell-cell communications, we used CellPhoneDB (version 3.0.0, https://github.com/Teichlab/cellphonedb) to define these ligand-receptor pairs across cell types and across predisposes/immune cell clusters^36^. Ligand-receptor pairs for each two cell types or two clusters were explored. The ligand-receptor pairs with p-value less than 0.05 were defined as significance. BMP related ligand-receptors were collected from reported article and infer their interactions in preadipocytes individually.

### Flow cytometric analysis of SVF of iWAT

Inguinal adipose tissues were dissected. The lymph nodes embedded were peeled out mechanically dissociated by smashing on 100μm cell strainer (Falcon 352360). Meanwhile, the rest adipose tissue (AT) without LN were enzymatically dissociated with collagenase VIII. Fresh SVF isolated from iWAT were fixed with 10% calf serum (CS) in PBS for 30 min. Cells were washed with PBS with 0.2% CS and incubated with fluorochrome-conjugated antibodies against surface antigens in 2% CS- PBS. Stained cells were analyzed with a BD FACSVerse flow cytometer. The data was analyzed with FlowJo v10.7.0. T cells were identified as CD45+ CD3+, macrophages were as CD45+CD3−NK1.1−F4/80+, NKT were as CD45+F4/80+CD11c+, DCs were as CD45+CD3−NK1.1−F4/80−CD11c+, and B cells were as CD45+CD3−F4/80−CD11c−CD19+. The commercial antibodies used were CD45-BV605 (BD biosciences 30-F11), F4/80-PE-Cy7 (Biolegend BM8), NK1.1-PerCP-Cy5.5 (BD Biosciences PK136), CD3-FITC (Biolegend 17A2), CD11c-PE (Biolegend N418), and CD19-BV786 (BD Biosciences 1D3). All the antibodies were used at a 1: 200 dilution in 1% CS in PBS.

### Western blot

Homogenized mouse iWAT from one same litter were lysed in 2% sodium dodecyl sulfate (SDS) tris buffer (60 mM, pH6.8) containing protease inhibitor cocktail (Roche). 20–50 μg protein was separated by SDS-PAGE and then transferred onto a polyvinylidene fluoride (PVDF, 0.22 µm) membrane. The commercial antibodies used were as following: anti-β-actin (Sigma A1978), anti-UCP1 (Abcam ab234430), anti-ATGL (Cell Signaling Technology, 2138S), anti-HSL (Cell Signaling Technology 4107), anti-phospho-HSL (Cell Signaling Technology 4139), Anti-ACC (Cell Signaling Technology 3662), anti-FADS1 (abcam ab126706), anti-FADS2 (abcam ab232898), anti-SCD1 (Cell Signaling Technology 2794), anti-Glut4 (Cell Signaling Technology 2213), anti-PPARγ (Cell Signaling Technology 2430), anti-C/EBPβ (Cell Signaling Technology 3087) and anti-HSP90 (Santa cruz, sc-13119). Horseradish peroxidase­conjugated secondary antibodies: anti-mouse (Abcam ab6728), anti-Rabbit (Abcam ab6721). All the primary antibodies were used at a 1: 1000 dilution in 3% bovine serum albumin (BSA) in TBST. Secondary antibodies were used at a 1:8000 dilution in 5% non-fat milk in TBST. The uncropped images along with images with referring protein markers were shown in Supplementary Fig. S10.

### Histology

Dissected adipose tissue was fixed in 4% paraformaldehyde for 24 h at room temperature. Fixed tissues were embedded in paraffin, sectioned at 5 µm thickness, deparaffinized and rehydrated through graded concentrations of ethanol in water. Sections were then stained with hematoxylin and eosin (H&E) (Sigma-Aldrich). For immunohistochemistry (IHC) staining, sections were probed with primary antibody against UCP1 (Abcam ab234430, 1:500), and Mki67 (Abcam ab279653, 1:500) followed by biotinylated secondary antibody. Binding of second antibodies was visualized by using diaminobenzidine (DAB) chromogen A (Thermo Fisher).

### RNA sequencing of iWAT

Total RNA was purified from inguinal adipose tissue of mice at different ages. Each group had 3 mice. Uniquely indexed libraries were generated per sample with the NEBNext UltraTM RNA Library Prep Kit for Illumina (NEB,USA), according to the manufacturer’s instructions. Indexed libraries were sequenced using the Illumina Hiseq platform, generating 125 bp long paired-end reads, yielding a minimum of ~20 million total reads per sample. After assessing sequence quality, STAR was used to map reads to the mouse genome (mm10) with default setting values. Mapping efficiency was >95% in all experiments^55^. Then mapped reads were annotated and FPKM (Fragments per kilo base per million mapped reads) was calculated with genecode M19 annotation reference. protein coding genes with FPKM ≥ 1 were used for differential expression analysis with fold change 2 as cutoff. Differential expressing genes were used for gene ontology analysis in KEGG pathways. Pathways with *p* value < 0.05 were exported and visualized in Cytoscape (v.3.9.1, https://cytoscape.org/index.html) in which the node sizes and color presented number of mapped genes and *p* value, respectively^56^.

### Statistics and reproducibility

Data distribution was assumed to be normal but this was not formally tested. All data were analyzed using Graphpad Prism v8.0.2.263 and are represented in the figures as mean values ± SD. *P* values were determined by a two-tailed Student’s *t* test for comparing two groups. *P* values are indicated with *p < 0.05, **p < 0.01; ***p < 0.001 and ****p < 0.0001 on graphs. Where graphs are not labeled with an asterisk, any differences between the test groups and the control groups were non-significant. ‘n’ in the figure legends indicate the number of biologically independent replicates. Western blot and micrographs results are representative experiments of more than three biologically independent replicates.

### Reporting summary

Further information on research design is available in the Nature Portfolio Reporting Summary linked to this article.

### Supplementary information

Supplementary information

Description of Additional Supplementary Files

Supplementary data 1

Supplementary data 2

Supplementary data 3

Supplementary data 4

Supplementary data 5

Supplementary data 6

Supplementary data 7

reporting summary

## Data Availability

The raw data of RNA-sequence was deposited in GEO database under accession number GSE249814. Published dataset was downloaded from GEO (GSE128889)^18^. Numerical source data for all graphs can be found in supplementary data 7 file. Other data relate to the main results can be available upon request from authors: Dr. Shuwen Qian, E-mail: shuwenqian2013@163.com; Dr. Zhao Zhang, E-mail: zhaozhang@fudan.edu.cn.

## Code availability

Codes were uploaded in GitHub (https://github.com/GieroZcy/scRNAseq-WAT, 10.5281/zenodo.11442689). Code will be provided to academic researchers upon reasonable request.
