# Supplementary material for: A single-cell sequence analysis of mouse subcutaneous white adipose tissue reveals dynamic changes during weaning
Source: Commun Biol. 2024 Jun 29;7:787. doi: 10.1038/s42003-024-06448-3 (PMC11217364; doi:10.1038/s42003-024-06448-3)

Supplementary Materials for

A single-cell sequence analysis of mouse subcutaneous white adipose tissue reveals dynamic changes during weaning

Shuwen Qian[1, #], Chenyang Zhang[1, #], Yan Tang[1], Mengyuan Dai[2], Zhihui He[1], Hong Ma, Linyuan Wang, Qiqi Yang, Yang Liu, Wei Xu[2], Zhao Zhang[1,*], Qi-qun Tang[1, *].

[1]Key Laboratory of Metabolism and Molecular Medicine of the Ministry of Education, Department of Biochemistry and Molecular Biology of School of Basic Medical Sciences and Department of Endocrinology and Metabolism of Zhongshan Hospital, Fudan University, Shanghai 200032, China.
[2]Department of Immunology, Shanghai Medical College, Fudan University, Shanghai 200032, China.

# These authors contribute equally.
*****Correspondence to:

Qi-qun Tang, E-mail: qqtang@shmu.edu.cn

Zhao Zhang, E-mail: zhaozhang@fudan.edu.cn

This file includes Fig. S1-S10

Supplementary figures and figure legends

Fig. S1: Enrichments of differential expressed genes in mouse iWAT among different developmental stages.

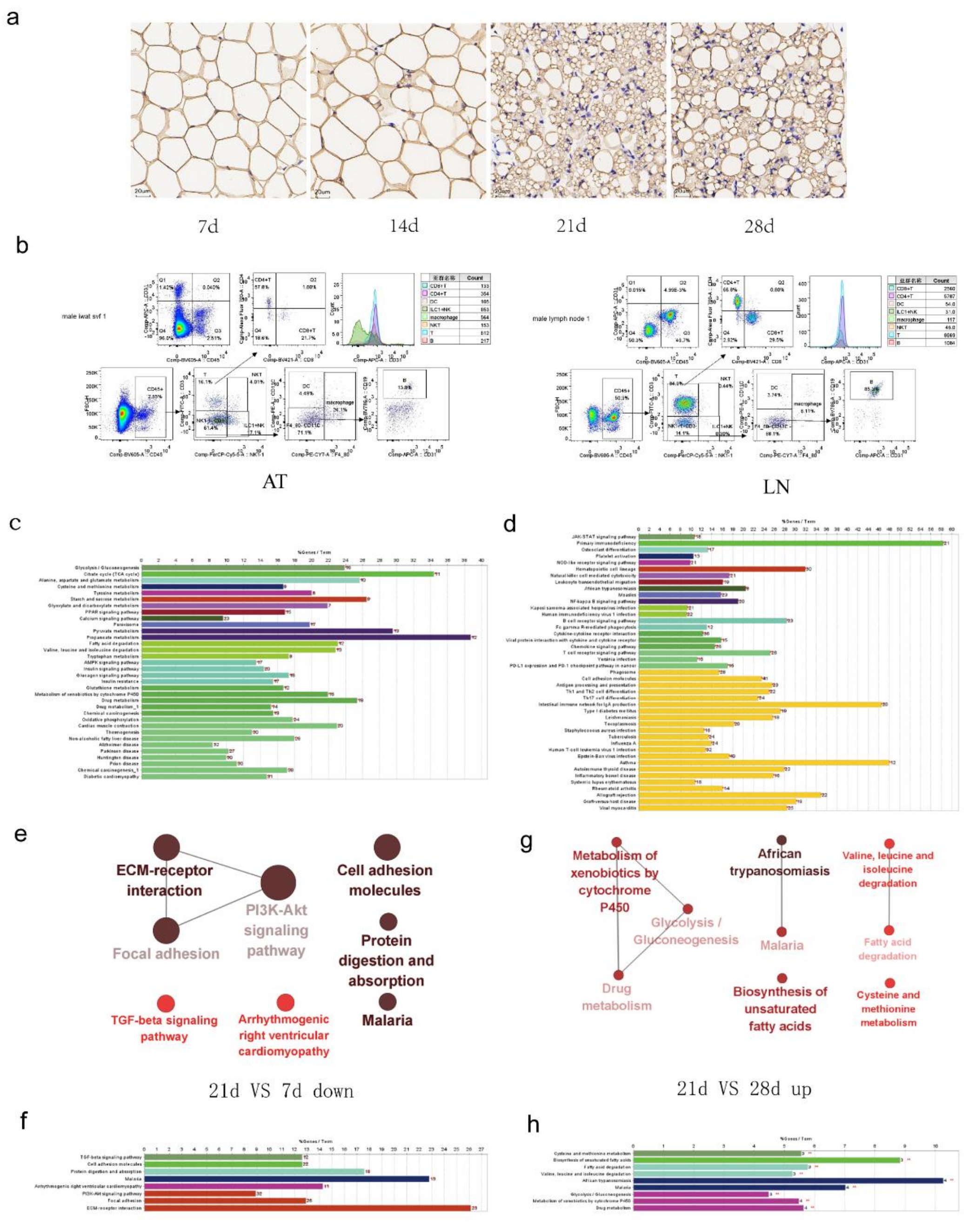

a IHC of iWAT with perilipin antibody to show lipid droplet structure. **b** Gating strategy of flowcytometry on subcutaneous adipose tissue (AT) excluding lymph node (LN) or LN. **c** Bar plot of KEGG pathway created by upregulated genes in iWAT at 21d as compared with 7d as determined by RNA-sequence. **d** Bar plot of KEGG pathway created by down regulated genes in iWAT at 21d as compared with 28d as determined by RNA-sequence. **e&f** RNA-sequence of iWAT and downregulated genes at 21d as compared with 7d were analysed by KEGG and ClueGo to show the pathways.

g&h RNA-sequence of iWAT and upregulated genes at 21d as compared with 28d were analysed by KEGG and ClueGo to show the pathways.

Fig. S2: Composition of heterogenous cell types in iWAT at developmental times.

a

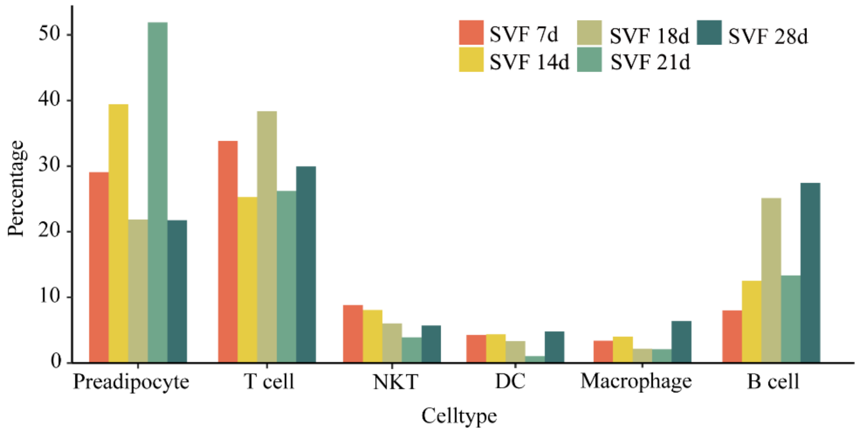

a Cell number percentages of all different cell types at different time points.

Fig. S3: Characteristic gene expressions of peadipocyte clusters.

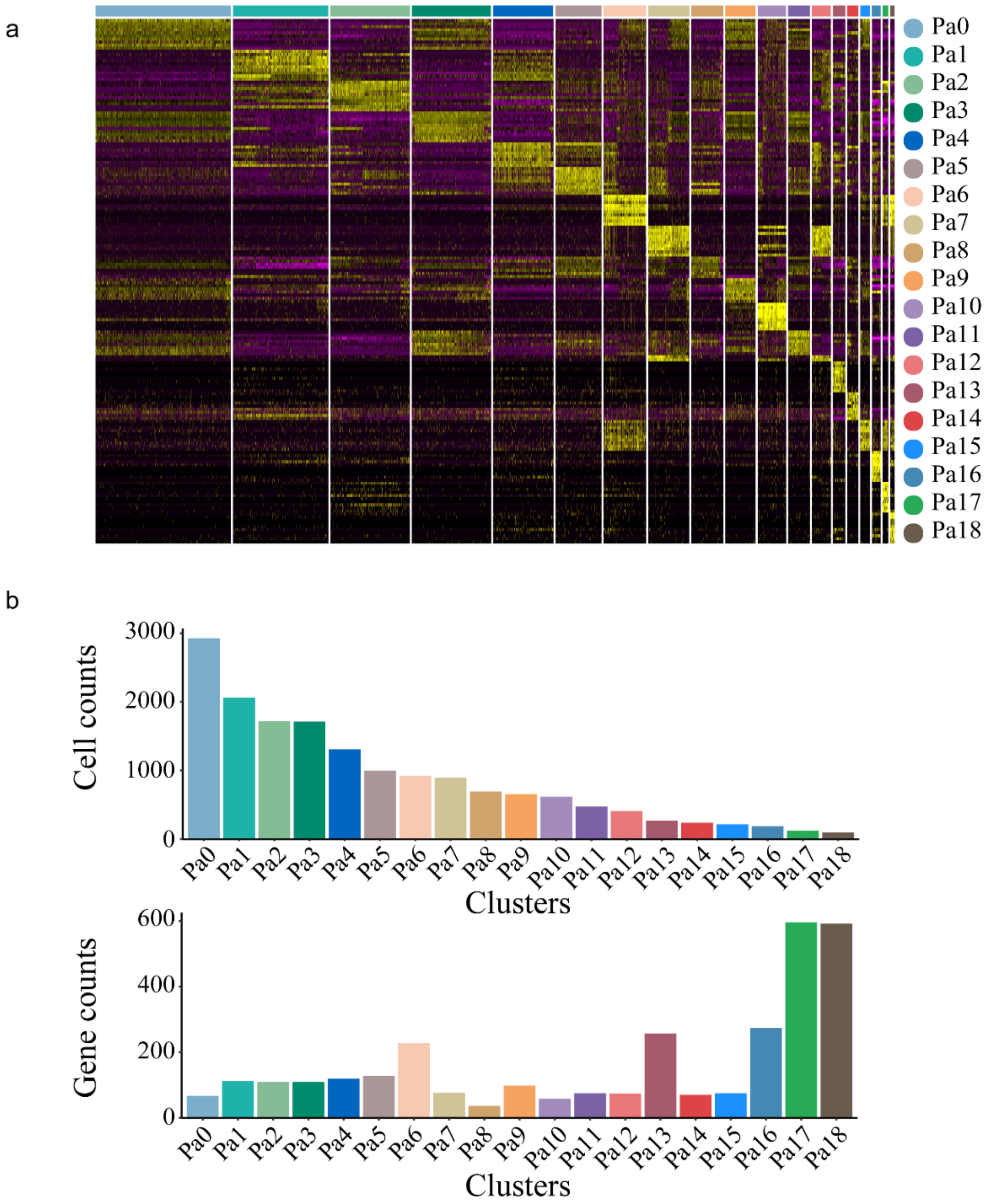

C

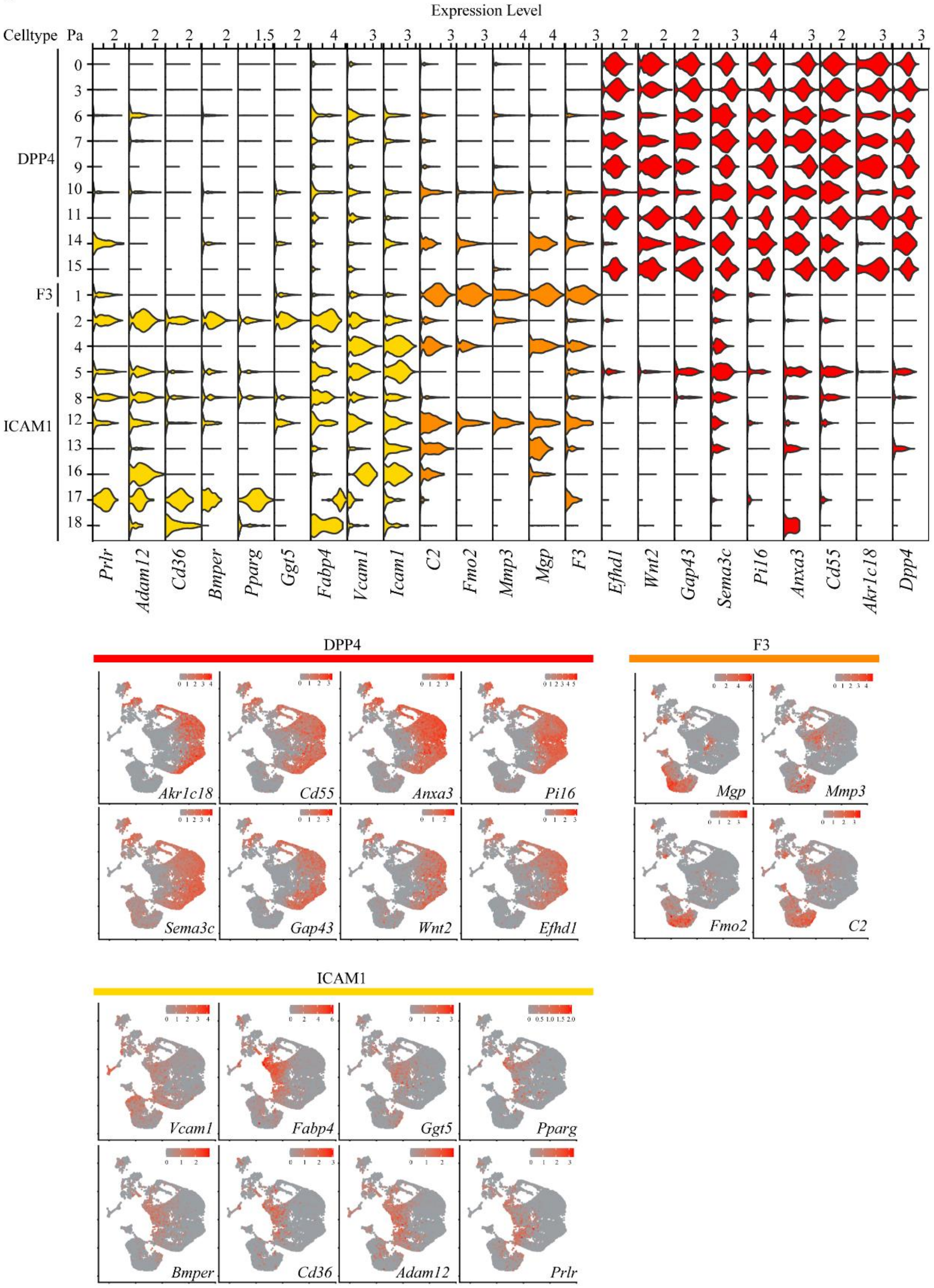
Expression Level
Celltype
Pa
DPP4
F3
ICAM1
Prlr
Adam12
Cd36
Bmper
Pparg
Ggt5
Fabp4
Vcam1
Icam1
C2
Fmo2
Mmp3
Mgp
F3
Efhd1
Wnt2
Gap43
Sema3c
Pi16
Anxa3
Cd55
Akr1c18
Dpp4
DPP4
Akr1c18
Cd55
Anxa3
Pi16
Sema3c
Gap43
Wnt2
Efhd1
F3
Mgp
Mmp3
Fmo2
C2
ICAM1
Vcam1
Fabp4
Ggt5
Pparg
Bmper
Cd36
Adam12
Prlr

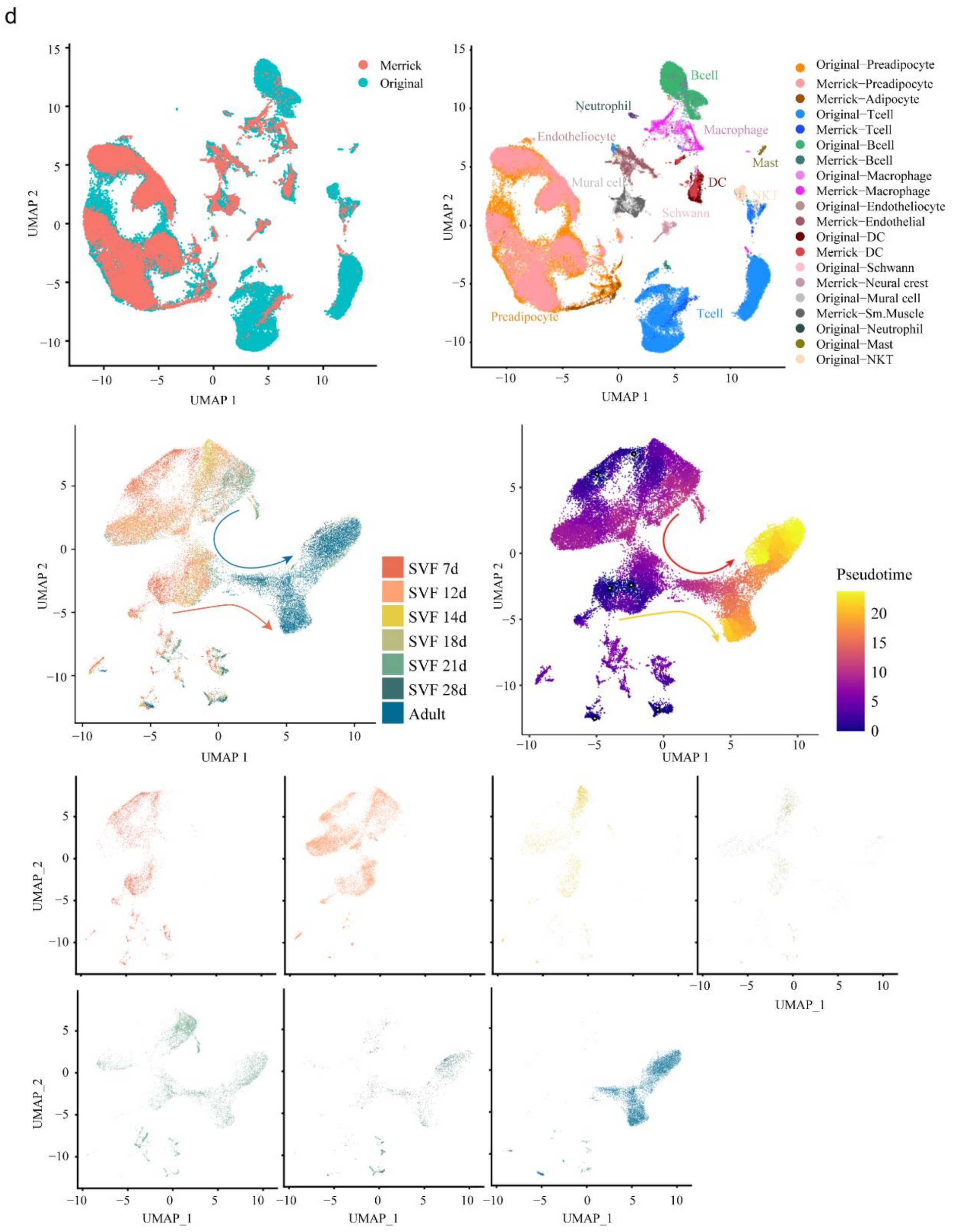

a Heat map of characteristic gene (top10) expressions in all preadipocyte clusters. **b** Cell counts (upper panel) and gene counts (lower panel) for all preadipocyte clusters. **c** Violin plot and UMAP projection show expression of marker genes in Pa clusters. **d** UMAP projection of cells integrating our and Merrick D’s data.

Fig. S4: Enrichment of genes in time-specific DPP4 preadipocytes.

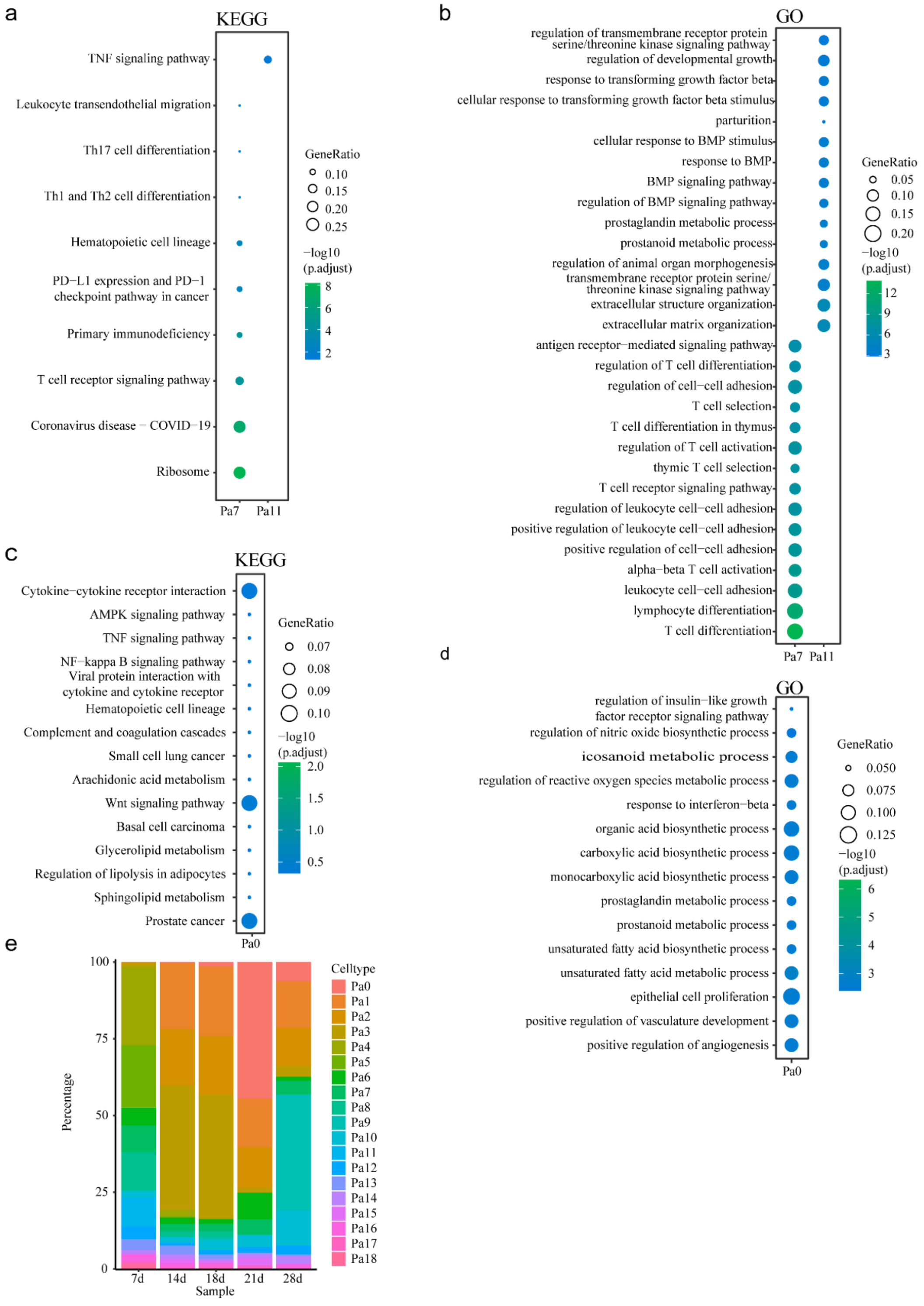

a KEGG analysis of characteristic genes in Pa7 and Pa11. **b** GO analysis of characteristic genes in Pa7 and Pa11. **c** KEGG analysis of characteristic genes in Pa0. **d** GO analysis of characteristic genes in Pa0. **e** Cell number percentages of all Pa0-P19 preadipocyte clusters.

Fig. S5: Enrichment of genes in time-specific ICAM1 preadipocytes.

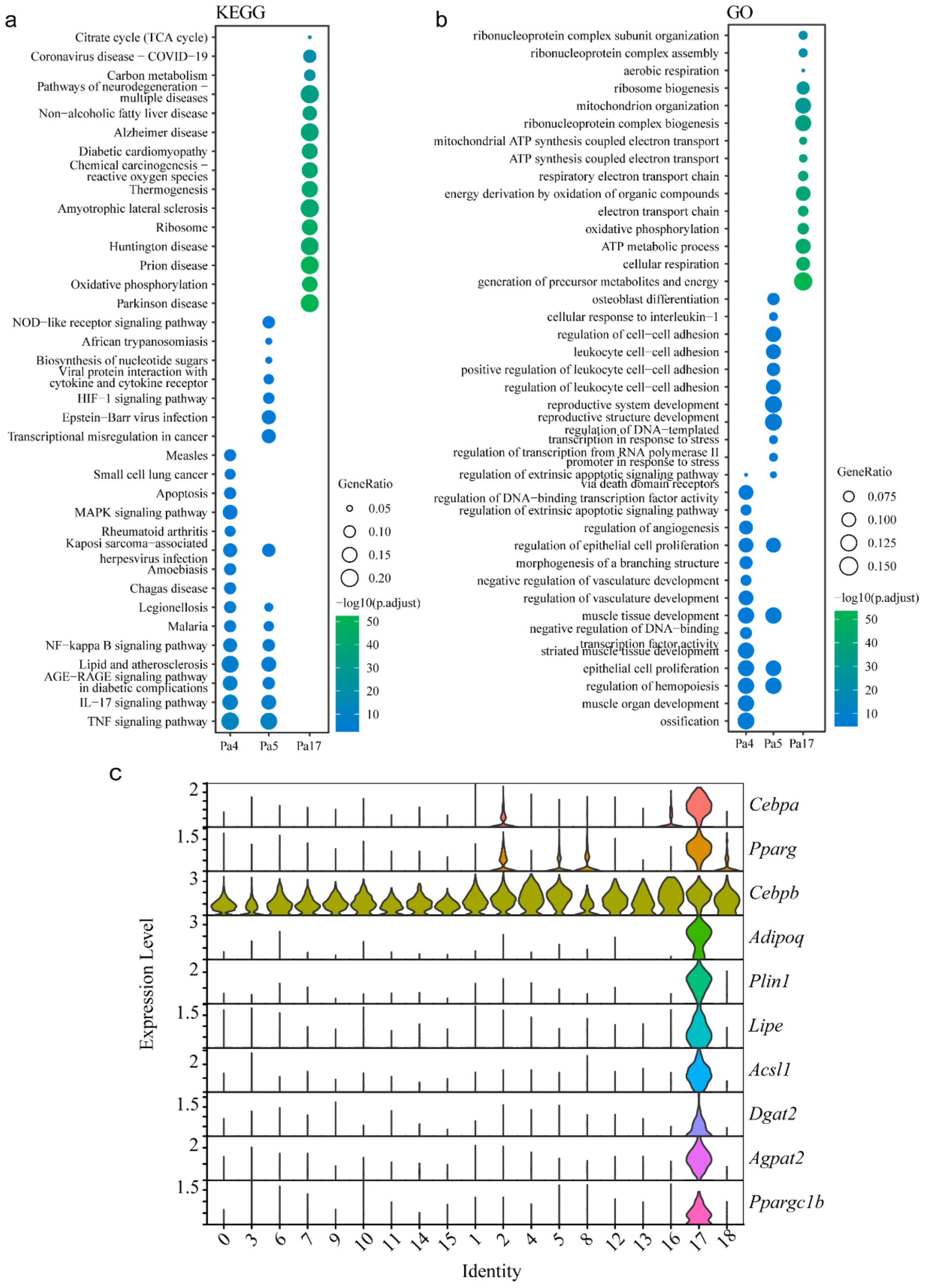

a KEGG analysis of characteristic genes in Pa4, Pa5 and Pa17. **b** GO analysis of characteristic genes in Pa4, Pa5 and Pa17. **c** Expression of some genes that are enriched in Pa17.

Fig. S6: Enrichment of genes in time-specific F3 preadipocytes.

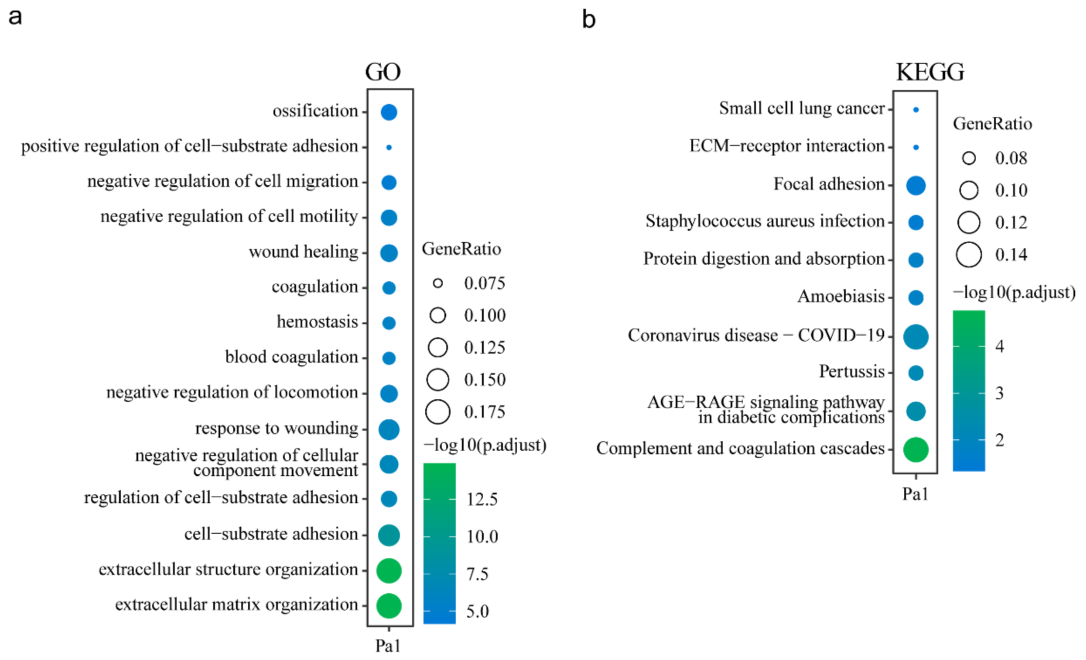

a KEGG analysis of characteristic genes in Pa1. **b** GO analysis of characteristic genes in Pa1.

Fig. S7: Enrichment of genes in the two proliferating preadipocytes.

a

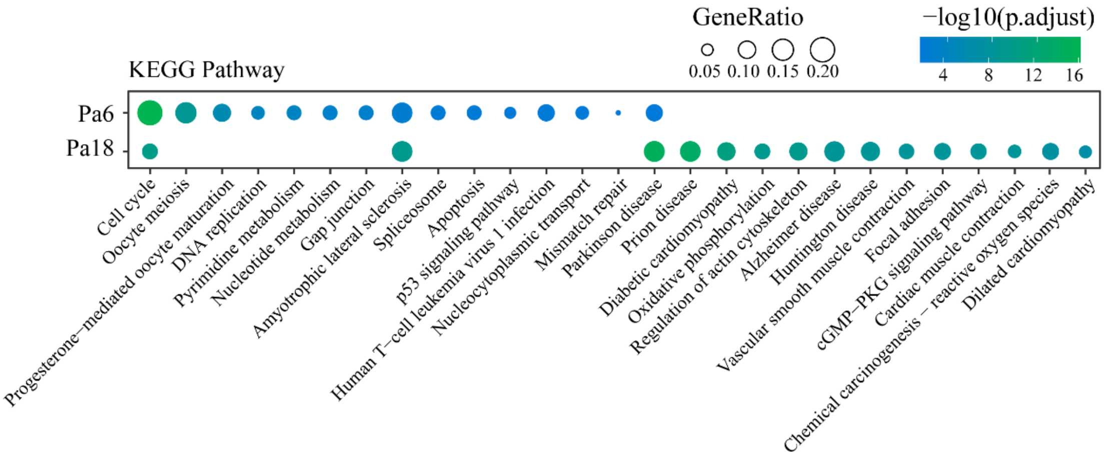

a KEGG analysis of Pa6 and Pa18, the two clusters with proliferative capacity. Top 15 pathways of all padj≤0.05.

Fig. S8: Developmental dynamics of immune cells in iWAT.

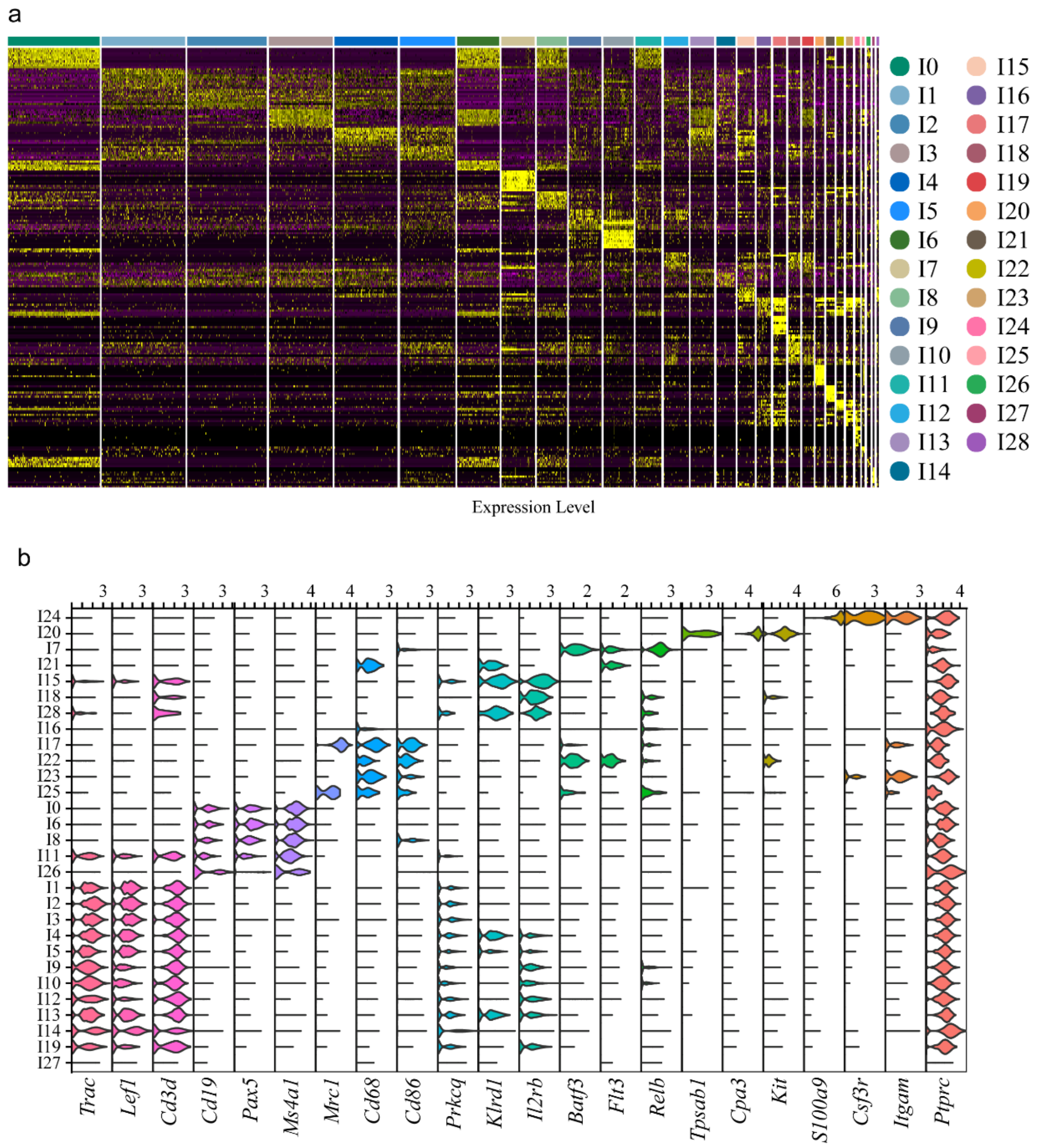

c

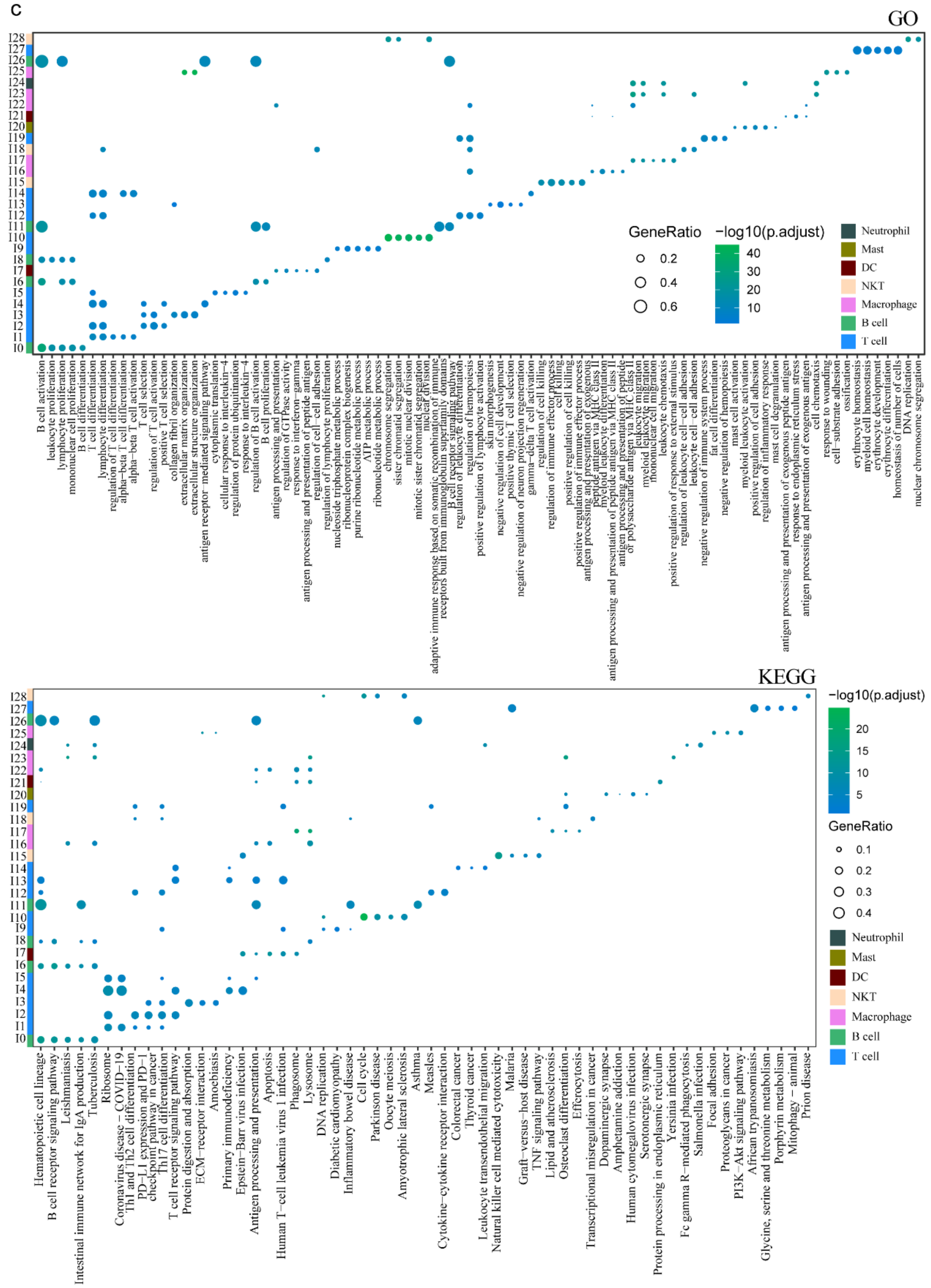
GO
GeneRatio
0.2
0.4
0.6
−log10(p.adjust)
40
30
20
10
Neutrophil
Mast
DC
NKT
Macrophage
B cell
T cell
I0
I1
I2
I3
I4
I5
I6
I7
I8
I9
I10
I11
I12
I13
I14
I15
I16
I17
I18
I19
I20
I21
I22
I23
I24
I25
I26
I27
I28
B cell activation
leukocyte proliferation
lymphocyte proliferation
mononuclear cell proliferation
B cell differentiation
T cell differentiation
regulation of T cell differentiation
lymphocyte differentiation
alpha−beta T cell differentiation
alpha−beta T cell activation
T cell selection
regulation of T cell activation
positive T cell selection
collagen fibril organization
extracellular matrix organization
extracellular structure organization
antigen receptor−mediated signaling pathway
cytoplasmic translation
cellular response to interleukin−4
regulation of protein ubiquitination
response to interleukin−4
regulation of B cell activation
B cell proliferation
antigen processing and presentation
regulation of GTPase activity
response to interferon−gamma
antigen processing and presentation of peptide antigen
regulation of cell−cell adhesion
regulation of lymphocyte proliferation
nucleoside triphosphate metabolic process
ribonucleoprotein complex biogenesis
purine ribonucleotide metabolic process
ATP metabolic process
ribonucleotide metabolic process
chromosome segregation
sister chromatid segregation
mitotic nuclear division
mitotic sister chromatid segregation
nuclear division
adaptive immune response based on somatic recombination of immune receptors built from immunoglobulin superfamily domains
B cell receptor signaling pathway
regulation of leukocyte differentiation
regulation of hemopoiesis
positive regulation of lymphocyte activation
skin morphogenesis
negative regulation of cell development
positive thymic T cell selection
negative regulation of neuron projection regeneration
gamma−delta T cell activation
regulation of cell killing
regulation of immune effector process
cell killing
positive regulation of cell killing
positive regulation of immune effector process
myeloid leukocyte migration
leukocyte migration
mononuclear cell migration
leukocyte chemotaxis
positive regulation of response to external stimulus
regulation of leukocyte cell−cell adhesion
leukocyte cell−cell adhesion
negative regulation of immune system process
fat cell differentiation
negative regulation of hemopoiesis
mast cell activation
myeloid leukocyte activation
positive regulation of cell adhesion
regulation of inflammatory response
mast cell degranulation
response to endoplasmic reticulum stress
antigen processing and presentation of exogenous antigen
cell chemotaxis
response to wounding
cell−substrate adhesion
ossification
erythrocyte homeostasis
myeloid cell homeostasis
erythrocyte development
erythrocyte differentiation
homeostasis of number of cells
DNA replication
nuclear chromosome segregation
KEGG
−log10(p.adjust)
20
15
10
5
GeneRatio
0.1
0.2
0.3
0.4
Neutrophil
Mast
DC
NKT
Macrophage
B cell
T cell
Hematopoietic cell lineage
B cell receptor signaling pathway
Leishmaniasis
Intestinal immune network for IgA production
Tuberculosis
Ribosome
Coronavirus disease − COVID−19
Th1 and Th2 cell differentiation
PD−L1 expression and PD−1 checkpoint pathway in cancer
Th17 cell differentiation
T cell receptor signaling pathway
Protein digestion and absorption
ECM−receptor interaction
Amoebiasis
Primary immunodeficiency
Epstein−Barr virus infection
Antigen processing and presentation
Apoptosis
Human T−cell leukemia virus 1 infection
Phagosome
Lysosome
DNA replication
Diabetic cardiomyopathy
Inflammatory bowel disease
Cell cycle
Parkinson disease
Oocyte meiosis
Amyotrophic lateral sclerosis
Asthma
Measles
Cytokine−cytokine receptor interaction
Colorectal cancer
Thyroid cancer
Leukocyte transendothelial migration
Natural killer cell mediated cytotoxicity
Malaria
Graft−versus−host disease
TNF signaling pathway
Lipid and atherosclerosis
Osteoclast differentiation
Efferocytosis
Transcriptional misregulation in cancer
Dopaminergic synapse
Amphetamine addiction
Human cytomegalovirus infection
Serotonergic synapse
Protein processing in endoplasmic reticulum
Yersinia infection
Fc gamma R−mediated phagocytosis
Salmonella infection
Focal adhesion
Proteoglycans in cancer
PI3K−Akt signaling pathway
African trypanosomiasis
Glycine, serine and threonine metabolism
Porphyrin metabolism
Mitophagy − animal
Prion disease

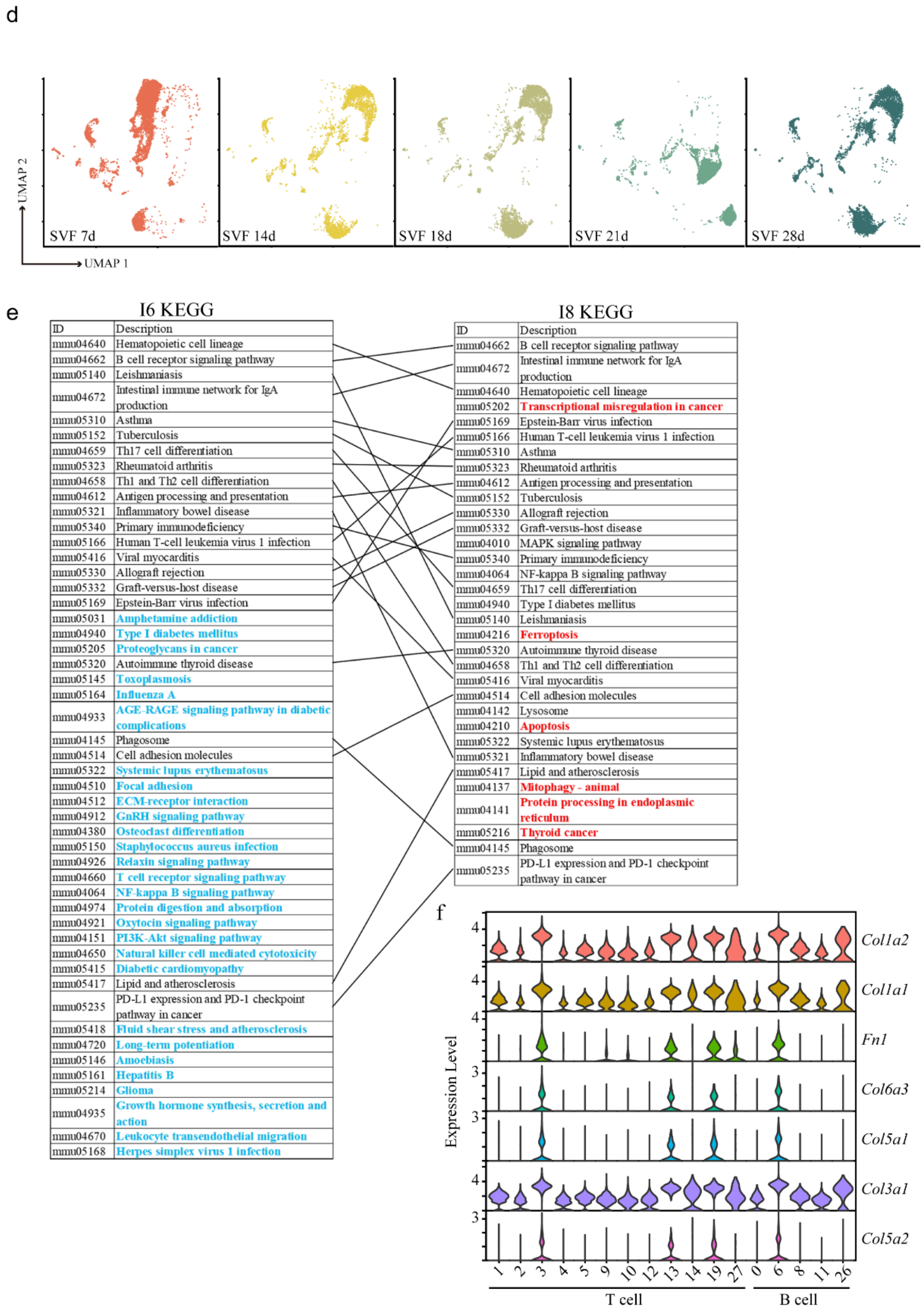

a Heat map of characteristic gene (top10) expressions in I0-I28 immune clusters. **b** Violin plot shows the express of marker genes for each immune cell cluster. **c** GO and KEGG analysis of genes in each immune cell clusters. **d** UMAP projections of immune cells clusters splitted by time points. **e** Comparison of KEGG pathways between I6 and I8 B cell clusters. **f** Expression of some extracellular matrix genes in T and B cells.

Fig. S9: Cell-cell interaction in iWAT determined by ligand-receptors.

a

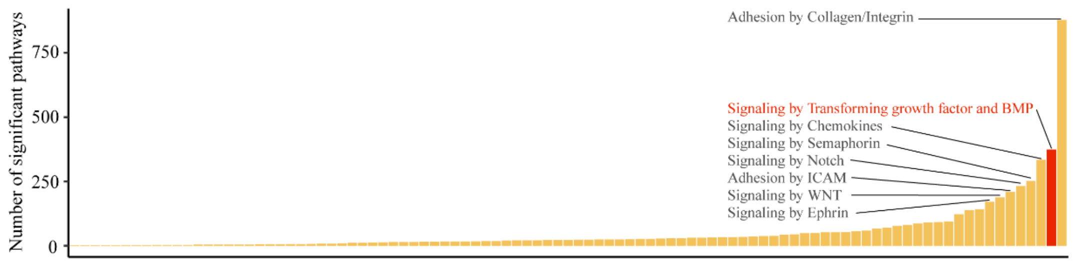

a The interactions between preadipocyte with other cell types in iWAT created by CellphoneDB with ranking of all families.

Fig. S10: RAW data for Western blot in Fig. 1.

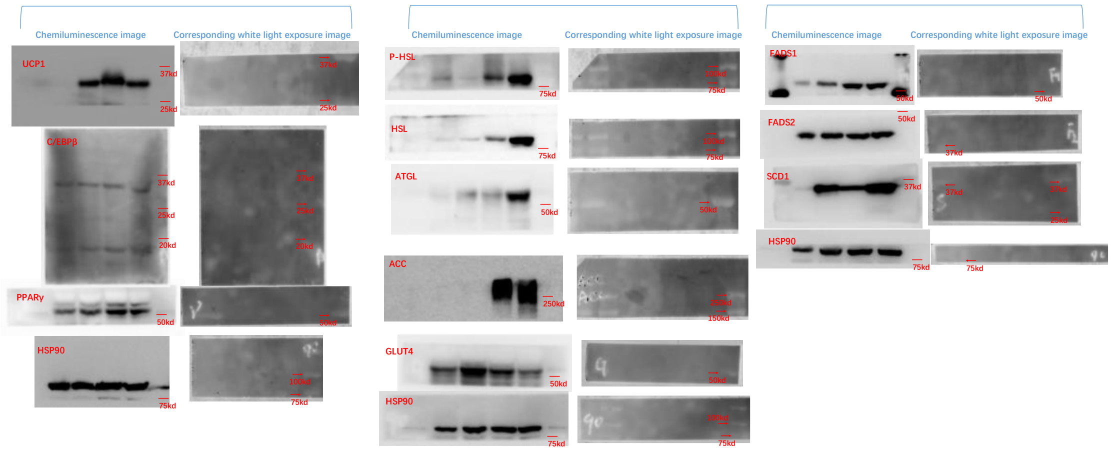

Supplement: Supplementary file 1 — Supplementary information [file 42003_2024_6448_MOESM1_ESM.pdf]
