# Supplementary material for: A single-cell sequence analysis of mouse subcutaneous white adipose tissue reveals dynamic changes during weaning
Source: Commun Biol. 2024 Jun 29;7:787. doi: 10.1038/s42003-024-06448-3 (PMC11217364; doi:10.1038/s42003-024-06448-3)
Supplement: Supplementary file 2 — Description of Additional Supplementary Files [file 42003_2024_6448_MOESM2_ESM.docx]

Description of Additional Supplementary Files

File name: Supplementary data 1

Description: Preadipocyte marker genes.

File name: Supplementary data 2

Description: Immune marker genes.

File name: Supplementary data 3

Description: GO of all preadipocytes subpopulations.

File name: Supplementary data 4

Description: KEGG of all preadipocytes subpopulations.

File name: Supplementary data 5

Description: GO of all immune cell subpopulations.

File name: Supplementary data 6

Description: KEGG of all immune cell subpopulations.

File name: Supplementary data 7

Description: Data behind all the bar graphs.

:

:

:

:

:

:

:
