# Supplementary material for: A single-cell sequence analysis of mouse subcutaneous white adipose tissue reveals dynamic changes during weaning
Source: Commun Biol. 2024 Jun 29;7:787. doi: 10.1038/s42003-024-06448-3 (PMC11217364; doi:10.1038/s42003-024-06448-3)
Supplement: Supplementary file 10 — reporting summary [file 42003_2024_6448_MOESM10_ESM.pdf]

nature research

Corresponding author(s): Qi-qun Tang

Last updated by author(s): May 17, 2024

Reporting Summary

Nature Research wishes to improve the reproducibility of the work that we publish. This form provides structure for consistency and transparency in reporting. For further information on Nature Research policies, see our Editorial Policies and the Editorial Policy Checklist.

Statistics

For all statistical analyses, confirm that the following items are present in the figure legend, table legend, main text, or Methods section.

n/a	Confirmed	
☐	☒	The exact sample size (n) for each experimental group/condition, given as a discrete number and unit of measurement
☐	☒	A statement on whether measurements were taken from distinct samples or whether the same sample was measured repeatedly
☐	☒	The statistical test(s) used AND whether they are one- or two-sided<br>*Only common tests should be described solely by name; describe more complex techniques in the Methods section.*
☒	☐	A description of all covariates tested
☒	☐	A description of any assumptions or corrections, such as tests of normality and adjustment for multiple comparisons
☐	☒	A full description of the statistical parameters including central tendency (e.g. means) or other basic estimates (e.g. regression coefficient) AND variation (e.g. standard deviation) or associated estimates of uncertainty (e.g. confidence intervals)
☐	☒	For null hypothesis testing, the test statistic (e.g. F, t, r) with confidence intervals, effect sizes, degrees of freedom and P value noted<br>*Give P values as exact values whenever suitable.*
☒	☐	For Bayesian analysis, information on the choice of priors and Markov chain Monte Carlo settings
☒	☐	For hierarchical and complex designs, identification of the appropriate level for tests and full reporting of outcomes
☒	☐	Estimates of effect sizes (e.g. Cohen's d, Pearson's r), indicating how they were calculated

Our web collection on statistics for biologists contains articles on many of the points above.

Software and code

Policy information about availability of computer code

Data collection	Single cell transcriptome capturation, Library construction and sequencing were created by 10 × genomics
Data analysis	Graphpad Prism, Image J, Cytoscape, 10X Genomics Cell Ranger, Seurat

For manuscripts utilizing custom algorithms or software that are central to the research but not yet described in published literature, software must be made available to editors and reviewers. We strongly encourage code deposition in a community repository (e.g. GitHub). See the Nature Research guidelines for submitting code & software for further information.

Data

Policy information about availability of data

All manuscripts must include a data availability statement. This statement should provide the following information, where applicable:

- Accession codes, unique identifiers, or web links for publicly available datasets
- A list of figures that have associated raw data
- A description of any restrictions on data availability

The authors affirm that all raw data will be available in the supplementary materials, RNA sequence data is deposit in GEO database (GSE249814).

Field-specific reporting

Please select the one below that is the best fit for your research. If you are not sure, read the appropriate sections before making your selection.

☒ Life sciences ☐ Behavioural & social sciences ☐ Ecological, evolutionary & environmental sciences

For a reference copy of the document with all sections, see nature.com/documents/nr-reporting-summary-flat.pdf

Life sciences study design

All studies must disclose on these points even when the disclosure is negative.

Sample size	Mouse experiments: 5-8 in each group. RNA-sequence: 3 samples in each group; Single-cell RNA Sequence: SVF cells at each time point were pooled from 2 mice.
Data exclusions	No data was excluded from the analysis.
Replication	Biological repeats were performed for each study.
Randomization	For experiments using mice , groups were determined by developmental times.
Blinding	Investigators were not blinded to mouse age during experiments.

Reporting for specific materials, systems and methods

We require information from authors about some types of materials, experimental systems and methods used in many studies. Here, indicate whether each material, system or method listed is relevant to your study. If you are not sure if a list item applies to your research, read the appropriate section before selecting a response.

Materials & experimental systems

n/a	Involved in the study	
☐	☒	Antibodies
☒	☐	Eukaryotic cell lines
☒	☐	Palaeontology and archaeology
☐	☒	Animals and other organisms
☒	☐	Human research participants
☒	☐	Clinical data
☒	☐	Dual use research of concern

Methods

n/a	Involved in the study	
☒	☐	ChIP-seq
☐	☒	Flow cytometry
☒	☐	MRI-based neuroimaging

Antibodies

Antibodies used	All the antibodies are commercially available, and the names of suppliers are described in method.
Validation	Validation is available on the manufacturer's website for use in mouse tissues

Animals and other organisms

Policy information about studies involving animals; ARRIVE guidelines recommended for reporting animal research

Laboratory animals	All the mice were of C57/B6L background. Animals are male mice unless otherwise stated. Age of the mice are described in the methods.
Wild animals	Not applicable.
Field-collected samples	Not applicable.
Ethics oversight	All studies were approved by the Animal Care and Use Committee of Shanghai Medical College, Fudan University.

Note that full information on the approval of the study protocol must also be provided in the manuscript.

Flow Cytometry

Plots

Confirm that:

- [x] The axis labels state the marker and fluorochrome used (e.g. CD4-FITC).
- [x] The axis scales are clearly visible. Include numbers along axes only for bottom left plot of group (a 'group' is an analysis of identical markers).
- [ ] All plots are contour plots with outliers or pseudocolor plots.
- [x] A numerical value for number of cells or percentage (with statistics) is provided.

Methodology

Sample preparation	Fresh SVF isolated from iWAT were blocked with 10⍰ calf serum (CS) in PBS for 30 minutes. Cells were washed with PBS and incubated with fluorochrome-conjugated antibodies against surface antigens in 2⍰ CS- PBS.
Instrument	Stained cells were analyzed with a BD FACSVerse flow cytometer.
Software	The data was analyzed with FlowJo.
Cell population abundance	Target cell populations were determined by positive surface marker staining.
Gating strategy	Immune cells were identified from CD45+ cells, and preadipocytes were identified from CD45- cells.

- [x] Tick this box to confirm that a figure exemplifying the gating strategy is provided in the Supplementary Information.
